# The western painted turtle genome, a model for the evolution of extreme physiological adaptations in a slowly evolving lineage

**DOI:** 10.1186/gb-2013-14-3-r28

**Published:** 2013-03-28

**Authors:** H Bradley Shaffer, Patrick Minx, Daniel E Warren, Andrew M Shedlock, Robert C Thomson, Nicole Valenzuela, John Abramyan, Chris T Amemiya, Daleen Badenhorst, Kyle K Biggar, Glen M Borchert, Christopher W Botka, Rachel M Bowden, Edward L Braun, Anne M Bronikowski, Benoit G Bruneau, Leslie T Buck, Blanche Capel, Todd A Castoe, Mike Czerwinski, Kim D Delehaunty, Scott V Edwards, Catrina C Fronick, Matthew K Fujita, Lucinda Fulton, Tina A Graves, Richard E Green, Wilfried Haerty, Ramkumar Hariharan, Omar Hernandez, LaDeana W Hillier, Alisha K Holloway, Daniel Janes, Fredric J Janzen, Cyriac Kandoth, Lesheng Kong, AP Jason de Koning, Yang Li, Robert Literman, Suzanne E McGaugh, Lindsey Mork, Michelle O'Laughlin, Ryan T Paitz, David D Pollock, Chris P Ponting, Srihari Radhakrishnan, Brian J Raney, Joy M Richman, John St John, Tonia Schwartz, Arun Sethuraman, Phillip Q Spinks, Kenneth B Storey, Nay Thane, Tomas Vinar, Laura M Zimmerman, Wesley C Warren, Elaine R Mardis, Richard K Wilson

**Affiliations:** 1 Department of Ecology and Evolutionary Biology, University of California, Los Angeles, Los Angeles, CA 90095-1606, USA; 2 La Kretz Center for California Conservation Science, Institute of the Environment and Sustainability, University of California, Los Angeles, Los Angeles, CA 90095-1496, USA; 3 The Genome Institute, Washington University School of Medicine, Campus Box 8501, 4444 Forest Park Avenue, St Louis, MO 63108, USA; 4 Department of Biology, Saint Louis University, St Louis, MO 63103, USA; 5 College of Charleston Biology Department and Grice Marine Laboratory, Charleston, SC 29424, USA; 6 Medical University of South Carolina College of Graduate Studies and Center for Marine Biomedicine and Environmental Sciences, Charleston, SC 29412, USA; 7 Department of Biology, University of Hawaii at Manoa, Honolulu, HI 96822, USA; 8 Department of Ecology, Evolution, and Organismal Biology, Iowa State University, Ames, IA 50011, USA; 9 Faculty of Dentistry, Life Sciences Institute University of British Columbia, Vancouver BC, Canada; 10 Benaroya Research Institute at Virginia Mason, Seattle, WA 98101 USA; 11 Department of Biology and Institute of Biochemistry, Carleton University, Ottawa, ON, Canada K1S 5B6, Canada; 12 School of Biological Sciences, Illinois State University, Normal, IL 61790, USA; 13 Department of Biological Sciences, Life Sciences Building, University of South Alabama, Mobile, AL 36688-0002, USA; 14 Research Computing, Harvard Medical School, Boston, MA 02115, USA; 15 Department of Biology, University of Florida, Gainesville, FL 32611 USA; 16 Gladstone Institute of Cardiovascular Disease, San Francisco, CA 94158, USA; 17 Cardiovascular Research Institute and Department of Pediatrics, University of California, San Francisco, San Francisco, CA 94158, USA; 18 Department of Cell and Systems Biology, University of Toronto, Toronto, ON, Canada M5S 3G5, Canada; 19 Department of Cell Biology, Duke University Medical Center, Durham, NC 27710, USA; 20 Department of Biochemistry and Molecular Genetics, University of Colorado School of Medicine, Aurora, CO 80045, USA; 21 Department of Biology, University of Texas at Arlington, Arlington, TX 76019, USA; 22 Department of Organismic and Evolutionary Biology, Harvard University, Cambridge, MA 02138, USA; 23 Museum of Comparative Zoology and Department of Organismic and Evolutionary Biology, Harvard University, Cambridge, MA 02138, USA; 24 Baskin School of Engineering University of California, Santa Cruz Santa Cruz, CA 95064, USA; 25 MRC Functional Genomics Unit, Department of Physiology, Anatomy and Genetics, Henry Wellcome Building of Gene Function, University of Oxford, Oxford, OX13PT, UK; 26 Cancer Research Program, Rajiv Gandhi Centre for Biotechnology, Poojapura, Thycaud P.O, Thiruvananthapuram, Kerala 695014, India; 27 FUDECI, Fundación para el Desarrollo de las Ciencias Físicas, Matemáticas y Naturales. Av, Universidad, Bolsa a San Francisco, Palacio de Las Academias, Caracas, Venezuela; 28 Biology Department, Duke University, Durham, NC 27708, US; 29 Bioinformatics and Computational Biology Laboratory, Iowa State University, Ames, IA 50011, USA; 30 Center for Biomolecular Science and Engineering, School of Engineering, University of California Santa Cruz (UCSC), Santa Cruz, CA 95064, USA; 31 Faculty of Mathematics, Physics and Informatics, Comenius University, Mlynska Dolina, Bratislava 84248, Slovakia

**Keywords:** Amniote phylogeny, anoxia tolerance, chelonian, freeze tolerance, genomics, longevity, phylogenomics, physiology, turtle, evolutionary rates

## Abstract

**Background:**

We describe the genome of the western painted turtle, *Chrysemys picta bellii*, one of the most widespread, abundant, and well-studied turtles. We place the genome into a comparative evolutionary context, and focus on genomic features associated with tooth loss, immune function, longevity, sex differentiation and determination, and the species' physiological capacities to withstand extreme anoxia and tissue freezing.

**Results:**

Our phylogenetic analyses confirm that turtles are the sister group to living archosaurs, and demonstrate an extraordinarily slow rate of sequence evolution in the painted turtle. The ability of the painted turtle to withstand complete anoxia and partial freezing appears to be associated with common vertebrate gene networks, and we identify candidate genes for future functional analyses. Tooth loss shares a common pattern of pseudogenization and degradation of tooth-specific genes with birds, although the rate of accumulation of mutations is much slower in the painted turtle. Genes associated with sex differentiation generally reflect phylogeny rather than convergence in sex determination functionality. Among gene families that demonstrate exceptional expansions or show signatures of strong natural selection, immune function and musculoskeletal patterning genes are consistently over-represented.

**Conclusions:**

Our comparative genomic analyses indicate that common vertebrate regulatory networks, some of which have analogs in human diseases, are often involved in the western painted turtle's extraordinary physiological capacities. As these regulatory pathways are analyzed at the functional level, the painted turtle may offer important insights into the management of a number of human health disorders.

## Background

Turtles (also known as chelonians or Testudines) are an enigma. As the vertebrate paleontologist Alfred Romer noted half a century ago, 'The chelonians are the most bizarre, and yet in many respects the most conservative, of reptilian groups. Because they are still living, turtles are commonplace objects to us; were they entirely extinct, [they] would be a cause for wonder'[1]. From the Triassic to the present, turtles have been morphologically conservative, and even the earliest turtles [2] are instantly recognizable. The living crown group of turtles extends back at least 210 million years [3] and is characterized by a number of unique morphological and physiological features. Besides their distinctive shell, turtles have extremely long lifespans, are often reproductively active at very advanced ages, often determine sex by the temperature at which eggs incubate, are the most anoxia-tolerant tetrapods known, and have the capacity in some species to freeze nearly solid, thaw, and survive with negligible tissue damage. The western painted turtle genome harbors a wealth of information on the genetic basis of these and other adaptations that characterize this unique vertebrate lineage.

Two of the great physiological challenges to vertebrate survival are hypoxia and cold tolerance. Particularly for temperate ectotherms like the western painted turtle, the two are closely linked, because winter hibernation often occurs underwater in ice-locked ponds, and involves long periods with limited access to oxygen. The western painted turtle is capable of surviving, with no loss of physiological function, 4 months under conditions of exceptionally low oxygen availability at 3°C [4] and at least 30 h at 20ºC [5]. This anoxia tolerance, when combined with the ability to survive freezing of 50% body water [6], allows hatchling painted turtles to endure long winters in their nests across the northern part of their range in North America. It also provides an unprecedented model to study natural mechanisms that protect the heart and brain from hypoxia-induced injury. Cardiac infarct and cerebral stroke are the first and third leading causes of death in the United States [7], and while conventional therapies continue to extend human lifespan, progress in improving outcomes from these conditions has been limited. Our genomic analyses indicate that painted turtles frequently achieve their extreme physiological capacities, at least in part, using conserved amniote molecular pathways; functional analyses of these pathways across turtles with varying physiological capacities thus may provide important insights for human disease prevention.

## Results and discussion

### Reference genome

We sequenced the nuclear genome of a single female western painted turtle, *Chrysemys p. bellii*, that we field-collected from southern Washington, using a combination of next-generation whole genome shotgun and Sanger-based BAC end reads (see Materials and Methods, *Sequencing and Assembly*, Additional file 1, Tables S1, S2). The assembly averages 18-fold coverage across 2.59 Gb with an N50 scaffold size of 5.2 Mb, and represents at least 93% of the genome. By all available measures, the assembled sequences have sufficient nucleotide and structural accuracy to provide a suitable template for initial analysis (see Materials and Methods, *Assembly Quality and Coverage Assessments*).

### Genome annotation

After soft masking the *C. p. bellii *genome with RepeatMasker [8], gene annotation was performed using the homology-based pipeline GPIPE [9-11] using a non-redundant protein set from human (Ensembl release 66), chicken (Ensembl release 66) and green anole (Ensembl release 66) as template. Based on the quality of the alignments with the template proteins, the conservation of exon boundaries and the absence of frame shifts and premature stop codons, we predicted a total of 21,796 protein-coding gene models in *C. p. bellii*, including 144,670 exons (average 6.63 exons per gene), and an average transcript size of 1,023 nucleotides (median 743 nucleotides). Using cDNAs obtained through 454 sequencing of libraries derived from brain, testes, ovaries, and trunk, we identified a total of 40,091 exons within 7,961 gene models to which cDNAs could be mapped.

### Repeat structure

Approximately 10% of the *C. p. bellii *assembly contains an abundance of transposable elements (TEs) that include nearly 80 distinct lineages of RNA-derived retrotransposons and DNA transposons, suggesting a long and dynamic history of clade-specific genomic diversification (see Additional file 1, Table S3, Additional file 2, Figures S1-S4). The western painted turtle exhibits intermediate TE copy number relative to birds and the lizard *Anolis*, and is rich in LTR elements including endogenous retroviruses, LINEs in the CR1 and RTE families, predominantly MIR-like SINEs, and DNA transposons (see Additional file 2, Figures S1-S3). These transposons include 385 *SPIN *elements in the hAT-Charlie family not previously detected by slot blot hybridization assays for seven turtle and four crocodilian species [12]. Consistent with the close evolutionary relationship between turtles and archosaurs (birds and crocodilians, see below), these elements and the overall genome have a GC content of 43% that is more similar to birds than *Anolis *[9,13] (see Materials and Methods, *Repeat Structure*, Additional file 1, Table S3, Additional file 2, Figures S1-S6). *Chrysemys p. bellii *also exhibits a moderate density of tandem repeats (1% genomic sequence coverage with an average density of 111 repeats per MB) with length and frequency distributions more similar to birds than to *Anolis *[13]. Overall, the repetitive landscape of *C. p. bellii *exhibits a substantial amount of lineage-specific evolution that distinguishes turtles from other major amniote taxa but exhibits some similarities to archosaurs, in keeping with their sister group relationship. Long generation times and a slow rate of molecular evolution may have facilitated the diversification of turtle repeats, potentially impacting both genomic stability and dynamics of transcriptome function [14-17].

### Isochore structure

**Figure 1:**
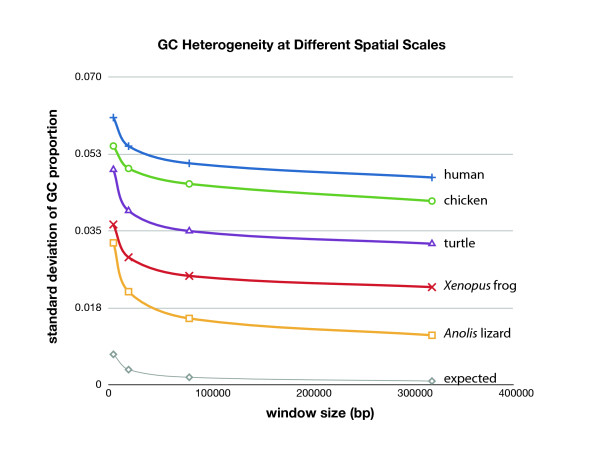
**Standard deviation of GC content at different spatial scales**. Genomes were partitioned into non-overlapping windows (5-, 20-, 80-, and 320-kb). As window size increases, variation in GC content naturally decreases. The western painted turtle exhibits a pattern consistent with high variation in nucleotide composition at smaller scales, rather than sustained isochoric variation at larger scales seen in mammals and birds. The expected pattern of decreasing standard variation assumes a compositionally homogeneous genome with a mean GC proportion of 0.41.

The presence of GC-rich isochores is a well-known feature of birds and mammals, but is a minor component of genomic structure in the lizard *Anolis*. The western painted turtle genome has an average GC proportion of 0.43, which is consistent with other amniotes (see Additional file 2, Figure S7). At a 3-kb scale, the standard deviation of GC content is 0.059, which is also intermediate among vertebrate genomes (see Additional file 2, Figure S7). The standard deviation of GC content in the western painted turtle is intermediate between those of the lizard *Anolis *and mammals/birds for sliding window sizes ranging from 5 kb to 320 kb (Figure 1), suggesting that the gene-rich isochores that characterize the endothermic birds and mammals are not as prominent a feature of the western painted turtle genome (see Materials and Methods, *Isochores*, Additional file 1, Table S4, Additional file 2, Figures S7-S9). For the western painted turtle, we found a weak but significant correlation between the GC content of protein-coding genes and their flanking sequence, indicating a slight, but potentially important relationship between genomic environment and the nucleotide composition of genes (see Additional file 2, Figure S8). We also found a slight negative relationship between the GC content and the length of intergenic sequences in the western painted turtle (not shown); thus, GC-rich regions tend to be more gene dense. This is a strong relationship in mammals and birds, but is non-existent in *Anolis*.

To examine the evolution of GC content in the context of the vertebrate phylogeny, we quantified GC content at third codon positions (GC3) using the 2,366 simple orthologs (1:1) identified from the OPTIC pipeline orthology predictions for zebrafish, pufferfish, chicken, zebrafinch, western painted turtle, green anole, platypus, mouse, and human (see Materials and Methods, *Identification of gene family expansion/contraction *for methods on determining gene homology). We used the program NHML (with default parameters) to estimate: (1) ancestral GC content; and (2) GC3*, the equilibrium GC content, which can be interpreted as the GC content toward which a lineage is evolving. Our results are consistent with trends from previous phylogenetic analyses of GC content [18,19], with the exception that chicken seems to be in equilibrium with regard to GC3. The western painted turtle shows a striking decrease in GC3 from its current value of 46.74% to a GC3* value of 38.90%, indicating an erosion of GC content that is also seen in *Anolis *(see Additional file 2, Figure S9) [18].

One mechanism that can contribute to this erosion is homogenization of recombination. Recombination is correlated with several evolutionary processes and genomic features. For instance, regions with higher recombination activity experience more efficient selection as well as higher GC content in mammals and birds. It stands to reason that genes with higher GC3 will have a lower lineage-specific dN/dS; that is, genes with higher GC content will experience more efficient selection. To test this, we divided up the genes from human, chicken, and western painted turtle into 'high GC3' and 'low GC3' genes based on the GC3 values of genes for each taxon. We then examined the distribution of dN/dS values between these two groups for each taxon. We expected, if recombination has a landscape similar to mammals and birds, that the 'high GC3' genes will have a lower lineage-specific dN/dS and 'low GC3' genes will have greater dN/dS values. We found this to be the case in human and chicken [18], indicating a heterogeneous recombination landscape (see Additional file 1, Table S4). In the painted turtle, we found that there is an even greater disparity in dN/dS between 'high GC3' and 'low GC3' genes than in human and chicken, indicating that an even more heterogeneous landscape exists in turtle. This may indicate that rather than a recombination-based mechanism driving GC content in turtle (for example, GC-biased gene conversion), mutational biases are playing an important role in the trajectory of GC3.

### Phylogeny and evolutionary rates

**Figure 2:**
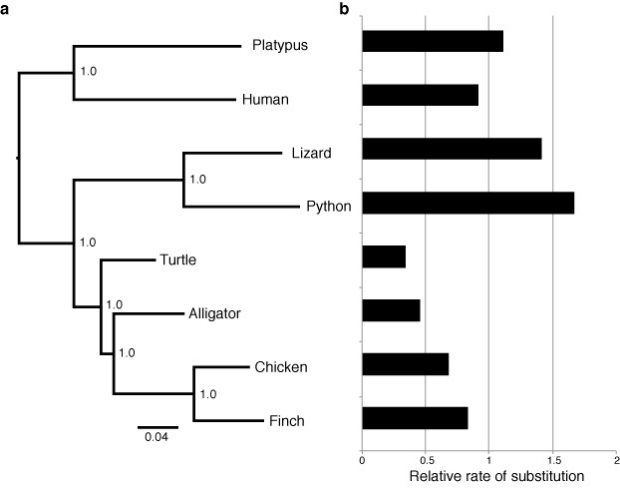
**A revised phylogeny of major amniote lineages and their rates of molecular evolution**. (**a**) Bayesian phylogram depicting the relationships of the eight primary amniote lineages, and their rates of molecular evolution. The phylogeny demonstrates the sister group relationship of turtle and archosaurs (allligator plus birds). The numbers at nodes denote posterior probabilities (all are at the maximum of 1.0). (**b**) The histogram shows the relative rate of substitution inferred for each lineage under a relaxed clock. For analysis details, see Materials and Methods, *Phylogeny and substitution rate*).

The phylogenetic position of turtles has remained one of the last unresolved problems in vertebrate evolutionary history, with recent hypotheses suggesting widely disparate placements [20,21]. Our phylogenetic analysis of 1,955 sets of rigorously screened gene orthologs (see Materials and Methods, *Multiple alignments and gene orthologs*) for eight vertebrate species (human, platypus, chicken, zebrafinch, anole, turtle, python, and alligator), analyzed separately or as a concatenated dataset, concur with two recent phylogenomic analyses [20,22] in placing turtles as the sister group to Archosauria with strong statistical support (Figure 2). Thus, based on independent, genome-scale analyses, the phylogenetic placement of turtles as well-nested within diapsid amniotes appears to be relatively secure.

We also estimated the relative rate of substitution in a smaller dataset that was designed to minimize missing data. This dataset comprised 309 orthologs that were identified in all eight species. Our analyses indicate that the turtle lineage has undergone a remarkable substitution-rate slowdown relative to other amniotes (Figure 2). Estimates of relative evolutionary rates under a relaxed molecular clock suggest that turtles have the slowest rate of substitution among the eight representative amniote lineages analyzed. Turtle genomes evolve at about one-third the rate seen in humans, and roughly one-fifth the rate of the fastest-evolving python lineage (see Materials and Methods, *Phylogeny and substitution rate*, Additional file 1, Tables S5-S6). Given the long generation time that characterizes turtles, our comparative analysis is consistent with the negative relationship between generation time and rate of molecular evolution found in reptiles [23] and other amniotes [24], although the observed slowdown in archosaurs and turtles may also suggest a broad, lineage-specific effect.

### Extreme anoxia tolerance in the painted turtle

Although all turtles can withstand anoxia for a few hours with no discernable tissue damage, the painted turtle is a candidate for the most extreme anoxia-tolerant tetrapod known. To explore the transcriptomic basis of this extreme anoxia tolerance, we assembled a gene expression profile by sequencing poly A-enriched RNA isolated from the ventricle (heart) and telencephalon (brain) of normoxic and anoxic (*n *= 4 turtles/group, 24 h at 19ºC) adult western painted turtles (see Materials and Methods, *Anoxic gene expression*). FPKM (Fragments per kilobase of exon model per million mapped fragments) values from 13,236 western painted turtle genes with human orthologs were interrogated (from a starting pre-filtering pool of 22,174 gene orthologs) and analyzed with ANOVA. Differential gene expression significantly increased in brain (19 genes) and heart (23 genes) (see Additional file 1, Tables S7, S8), mirroring previous work showing up-regulated gene expression in response to hypoxia in other vertebrate tissues, including many cancers.

The largest overall change in expression was in *APOLD1*, an apolipoprotein encoding gene whose transcript levels increased 128-fold in telencephalon and 19-fold in ventricle (see Additional file 1, Tables S7, S8; Additional file 2, Figure S10). *APOLD1 *expression moderately increases during hypoxia in human microvascular endothelial cell culture, although its exact function remains unclear [25]. Other highly differentially expressed genes (>10-fold; *FOS*, *JUNB*, *ATF3*, *PTGS2*, *BTG1/2*, and *EGR1*) encode proteins that, individually and in dimeric forms, have been implicated in the control of cellular proliferation, cancers, and tumor suppression [26-29]. The 30-fold increase in a gene orthologous to *SLC2A1 *(see Additional file 1, Table S8, Additional file 2, Figure S11), which encodes the glucose transporter GLUT-1, is also exceptional since deficiencies in membrane glucose transport underlie diabetes in humans. An understanding of the mechanism by which membrane GLUT-1 levels increase in the turtle would be a useful contribution to human diabetes research. Decreases in gene expression were fewer and found only in ventricle (see Additional file 1, Table S9; Additional file 2, Figure S12), but included decreases in *CDO*, which is important in regulating intracellular cysteine as well as levels of the endogenous metabolic depressant hydrogen sulfide [30,31], and genes involved in mRNA splicing (*SRSF5*) [32] and tumor proliferation (*MKNK2*) [33].

These analyses demonstrate the power of the western painted turtle as a model for the evolution of anoxia tolerance by regulatory changes utilizing broadly conserved vertebrate genes, including many genes that lead to pathogenesis in humans. Clearly, further study of the processes that link these regulatory changes to anoxia tolerance are a next important step. Although this is yet to be tested, we also note that the regulatory pathways that evolved in the western painted turtle could lead to the identification of targets for therapeutic intervention in human diseases involving hypoxic injury and possibly tumorigenesis.

### A novel microRNA associated with freeze tolerance in hatchling painted turtles

Freeze tolerance constitutes a second suite of physiological adaptations that are integral to winter survival for hatchling painted turtles and other species that overwinter in shallow terrestrial nests. Molecular adaptations that underlie natural freezing survival in *C. p. bellii *include strong metabolic rate depression, use of anaerobic metabolism (see *Extreme Anoxia Tolerance in the Painted Turtle*), and selective up-regulation of genes involved in key cellular processes [34].

**Figure 3:**
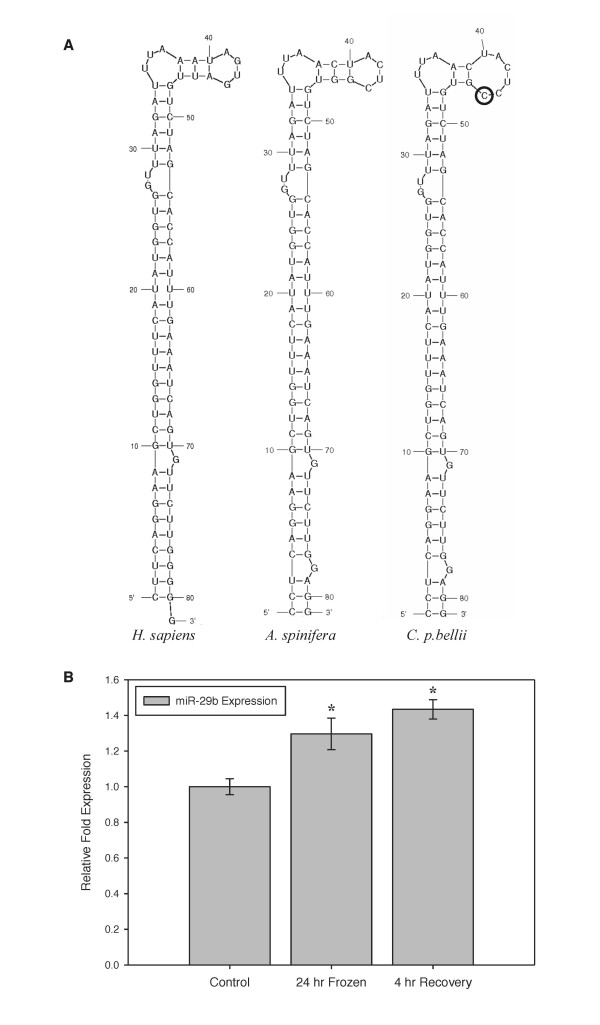
**Western painted turtle miR-29b and response to freezing**. (**a**) Nucleotide sequence and predicted secondary structure of pre-miR-29b transcripts from *H. sapiens*, *A. spinifera*, and *C. p. bellii *at 25 C. Nucleotide substitution which leads to differential terminal stem-loop formation that is unique to *C. p. bellii *is circled. (**b**) Relative expression levels of miR-29b as assessed by quantitative RT-PCR in liver samples of hatchling western painted turtles under control (5°C acclimated), 24 h frozen (at -2.5°C), or 4 h thawed (at 5°C) conditions. Data are means ± s.e.m. (*n *= 5 different animals). Parallel analysis of 5S rRNA found no significant changes between control and experimental conditions for this reference RNA. * Significantly different from the corresponding control (*P *<0.05).

Entrance into hypometabolism involves regulatory changes in multiple metabolic processes coordinated by extracellular stimuli that are readily induced and reversed to allow smooth transitions to and from the frozen state. MicroRNA regulation of mRNA transcripts meets these criteria and is involved in other models of stress-induced metabolic rate depression [35]. Using the western painted turtle genome, we retrieved the precursor sequence of miR-29b, a microRNA involved in DNA methylation and regulation of glucose transport [36,37] that is often associated with freeze and anoxia tolerance (see Materials and Methods, *Freeze tolerance*). Based on this sequence, the secondary structure of western painted turtle pre-miR-29b was predicted to contain a single nucleotide mutation (nuc-43) resulting in a larger terminal stem-loop compared to the less freeze tolerant turtle *Apalone spinifera *and *Homo sapiens*. Although the functional significance of this mutation is unknown, microRNAs are generally extremely conserved across vertebrates, and nucleotide structures that restrain the terminal loop region (as predicted for human and other turtles) can decrease the efficiency of Dicer processing of precursor microRNA transcripts in the range of 50% (Figure 3A) [38]. In addition to loop flexibility, slight alterations to loop structure and nucleotide sequence can influence interactions between pre-microRNA and terminal loop binding proteins, impacting processing efficiency. Consistent with the hypothesis that enhanced microRNA processing under low temperature stress facilitates freezing survival, quantitative RT-PCR (see Materials and Methods, *Freeze tolerance*) revealed a mild but statistically significant 1.3-fold increase in processed mature miR-29b levels in liver of hatchling turtles in response to 24 h freezing; expression was maintained and possibly increased during subsequent thawing (Figure 3B).

Although these results require additional functional analyses and are clearly preliminary, they point to future work on miR-29b as a potential candidate for freeze tolerance work on turtles with this physiological capacity. With refined genomic and comparative data across freeze tolerant and intolerant turtles, future studies of turtle freeze tolerance should help confirm or refute our interpretation that mutations in miR-29b are an important component of freeze tolerance in turtles.

### Tooth loss pseudogenization

**Figure 4:**
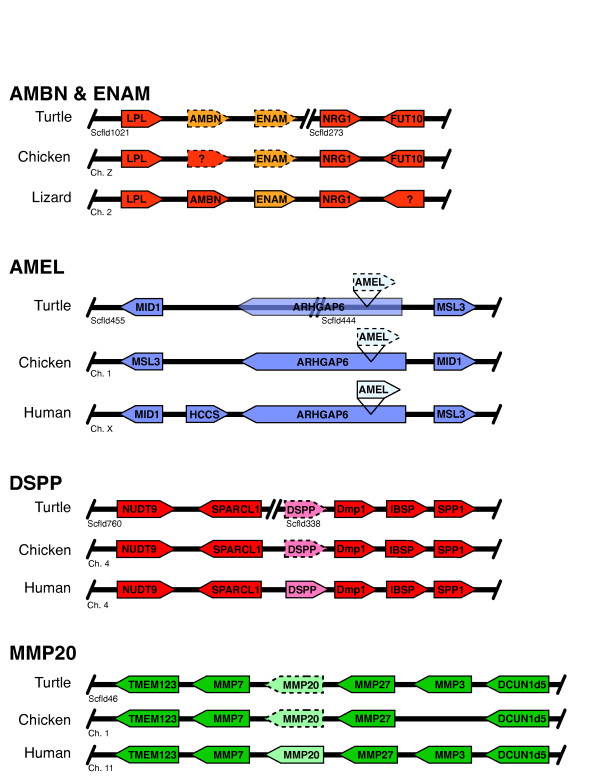
**Conserved syntenic regions containing tooth-specific genes across toothed (human, anole) and edentulous (turtle, chicken) vertebrates**. *AMBN *and *ENAM *are in a reptile-specific chromosomal region, precluding the use of human as a reference sequence for these genes. Dashed outlines indicate pseudogenization.

Turtles lost the ability to form teeth approximately 150-200 million years ago, making them the oldest extant edentulous lineage of tetrapods (birds lost teeth approximately 80-100 million years ago) [39]. Previous studies in birds and edentulous mysticete (baleen) whales demonstrated that tooth loss is closely associated with the pseudogenization and subsequent degradation of the tooth-specific genes enamelin (*ENAM*), amelogenin (*AMEL*), ameloblastin (*AMBN*), dentin sialophosphoprotein (*DSPP*), and enamelysin (*MMP20*) [40,41]. We identified the majority of turtle pseudo-exons in their chromosomally syntenic regions (see Materials and Methods, *Tooth loss*) when compared to other amniotes (Figure 4), consistent with the very slow rate of genomic change seen in chelonians (see Figure 2). Turtle *ENAM*, *AMEL*, and *MMP20 *all contain premature stop codons (exons 5, 3, and 2, respectively) in addition to highly degenerated sequences. *AMBN*, while somewhat more conserved, has a premature stop codon in exon 7. While *DSPP *exons 1 and 2 are relatively conserved, all subsequent exons were unidentifiable. Sequence identity scores between pseudogene exons identified in turtle and chicken were not significantly different from each other compared to their functional orthologs in crocodilians (see Materials and Methods, *Tooth loss*, Additional file 1, Tables S10, S11), even though turtles lost their teeth approximately 50-100 million year earlier.

This extremely conservative pattern of tooth-loss pseudogenization across amniotes is consistent with a single evolutionary origin (and regulatory network) of teeth, and suggests that the deterioration of this pathway evolved independently (that is, is homoplastic) in turtles, whales, and birds. This is also consistent with the fossil record, as early members of all three lineages are known to be toothed. However, concordant with their overall slow rate of molecular evolution, the tooth-specific genes in turtles have accumulated mutations at roughly half the rate of accumulation found in birds.

### The genomic basis of longevity in turtles

One of the defining features of turtles as a lineage is their extreme longevity (many species live 100 years or more), and we used the western painted turtle genome to investigate this quintessential chelonian feature. Based on previous work implicating the shelterin complex encoding genes in exceptional longevity in the naked mole rat [42], we evaluated (by BLAST searches of all available turtle sequence data including unplaced scaffolds, see Materials and Methods, *Aging and longevity*, Additional file 1, Table S12) the status of the shelterin complex in the western painted turtle genome. Even with this comprehensive search, we were unable to find orthologs for three of the five genes (*POT1*, *TERF2IP*, *TEP1*) in the western painted turtle. Given that *TEP1 *is also absent in birds, this result strongly suggests that turtles (and their sister group, the archosaurs) do not share this longevity mechanism with the naked mole rat.

We also examined genes that have apparently been lost in the western painted turtle (and were also absent in our searches of all other available turtle genomes) to investigate their relevance to aging based on their orthology to known aging-linked genes in model organisms [43]. Specifically, lowered activity of *ATP5O *in the nematode *C. elegans *increases longevity [44], while *PLCG2 *is a crucial intracellular signaling modulator and seems to be negatively affected by aging [45]. Although confirming the absence of genes is difficult with incompletely assembled genomes, the western painted turtle genome is at least 93% complete, and their absence in other turtle genomes is compelling (see Additional file 1, Table S12). Among these presumably missing genes, the lack of *ATP5O *(for which we found no hits in any turtle) and *PLCG2 *(where we found evidence for a total of six out of 30 exons across all turtles) may be important in the extraordinary longevity of turtles.

### Temperature-dependent sex determination/differentiation (TSD) genes

Since the first realization that many, but not all, turtles have TSD, turtles have become a model system for comparing the gene networks controlling genotypic sex determination (GSD) and TSD. Phylogenetic reconstruction indicates that the ancestral condition of sex determination in turtles and crocodilians was thermosensitive (TSD), and that GSD has re-evolved in several turtle lineages [46]. Although it is now clear that TSD and GSD each encompass multiple mechanisms whose divergence involves regulatory and structural evolution affecting the level of plasticity and canalization of vertebrate sexual development [47,48], it also remains the case that transitions between TSD and GSD have occurred many times, and that TSD is the ancestral condition in turtles. Genomic analyses of TSD and GSD turtles (and crocodilians) can provide important clues to help decipher the changes in genetic architecture that underlie these evolutionary transitions. Comparative analysis of genomes and transcriptomes from TSD turtles (*Chrysemys p*. *bellii*, *Chelydra serpentina*, *Trachemys scripta*) and the GSD softshell turtle *Apalone mutica *(all data produced by our group) from early through late embryonic stages revealed that virtually all of the known vertebrate genes involved in sexual differentiation are present in turtle genomes and active during sexual development (see Materials and Methods, *Sex determination/differentiation*, Additional file 1, Table S13).

**Figure 5:**
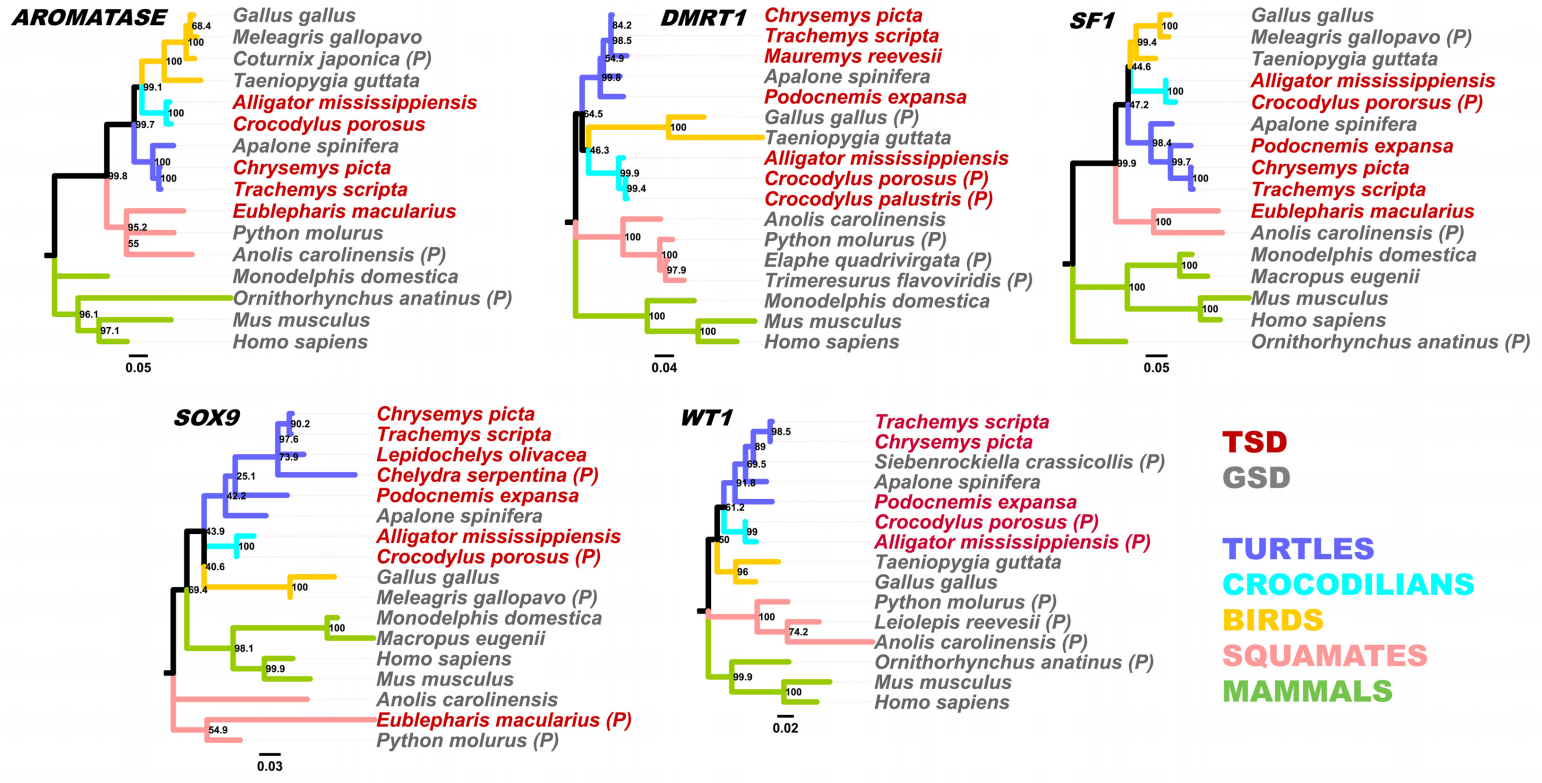
**Maximum likelihood estimates of the phylogenetic relationships among taxa for five genes involved in gonadogenesis**. Branch lengths are proportional to the number of substitutions per site; numbers at nodes are bootstrap proportions based on 500 pseudoreplicates. Colored branches denote the taxonomic group for each taxon. Tip font colors denote sex-determining mechanisms (red = TSD, gray = GSD). For all species, the full coding region was utilized except where only partial sequences were available, in which case the tip is denoted as (P).

We took a gene-tree reconstruction approach to examine the phylogenies of the coding regions of five key genes involved in the gonadogenesis regulatory network whose transcriptional responses have been studied in the western painted turtle (*WT1*, *SF1*, *SOX9*, *DMRT1*, and *AROMATASE *[48,49], Figure 5). Although the roles of these genes in the TSD/GSD transition remains incompletely understood, they are important in sexual differentiation in a variety of vertebrates including reptiles. Our primary goal was to ask whether these individual gene trees cluster taxa based on their phylogenetic relationships (as might be expected if independent TSD/GSD transitions have evolved that do not mask phylogeny) or on their TSD/GSD phenotype. Consistent with their phylogenetic relationships, our gene tree analyses generally placed the monophyletic set of turtle orthologs as the sister group to archosaurs (compare the relationships of turtles and crocodilians in Figure 2 with Figure 5), although in one case (*WT1*) TSD turtles and crocodilians were sister groups (Figure 5). However, within-turtle relationships of these five gene trees often resolve the GSD softshell *Apalone spinifera *as sister group to the remaining turtles, rather than in its generally established placement as sister to the remaining cryptodires [50]. It is well known that estimates of individual gene trees can differ from species trees for purely statistical reasons, and the inter-relationships of softshells to other turtles has been notoriously difficult to determine with molecular data [50-52].

Overall, there is no compelling evidence of clustering TSD and GSD turtles, or TSD and GSD vertebrates that is contrary to their phylogenetic relationships, suggesting that strong convergence at the molecular level has not occurred in these markers. Interestingly, dn/ds analysis revealed that the molecular evolution of these elements is driven overwhelmingly by purifying selection, with only few instances of neutral evolution between some closely related species pairs such as *Trachemys scripta *(TSC) and *Chrysemys p. bellii *(CPI) for *SF1*, *AROMATASE *and *Wt1*, TSC and *Apalone spinifera *(ASP) for *SF1*, ASP and CPI for *SF1*. Thus, these analyses indicate that the primary patterns of gene tree evolution in these loci associated with sex determination are driven by their organismal (phylogenetic) history rather than TSD/GSD functionality.

### Immune system genomics

Given the striking preponderance of expansions of immune function genes (see below), and their potential importance in the extended life spans of turtles, we characterized a large panel of immune-function genes in the western painted turtle genome. We aligned the *C. picta bellii *genome against a sequence database of approximately 3,000 immune-function related genes developed from a diverse set of 14 vertebrates ranging from lamprey to mammals (see Materials and Methods, *Immune system*). Blast searches of the *C. picta bellii *genome against this database resulted in the identification of 110 genes, 100 of which were confirmed with reciprocal alignments; 73 were also identified in either cDNA or predicted gene sequences (see Additional file 1, Table S14). The cDNA represented a small number of tissues/developmental stages, and 73/110 (66%) confirmation of expression is very encouraging.

The adaptive immune response of turtles is generally slower and less robust than its mammalian counterpart, and does not consistently demonstrate evidence of a memory response [53,54]. However, we identified several major components necessary for adaptive immunity and generation of immune memory including *CD4*, *MHCII*, and the immunoglobulin heavy chain locus (see Additional file 1, Table S14). Our analysis also demonstrates that the western painted turtle has a unique repertoire of toll-like receptors (TLRs), comprised of those found in amphibians, fish, birds, and mammals. This includes a TLR15-like receptor that has previously only been defined in birds, and is known to interact with bacterial pathogens including *Salmonella *[55] (see Additional file 1, Table S15). Given the delayed adaptive response and poor generation of immune memory, combined with their diverse set of TLRs, we predict that turtles should rely more heavily on the non-specific innate immune response to effectively recognize and initiate appropriate responses to pathogens. This initial response would be followed by a more moderate adaptive response that, because of the low specificity due to lack of immune memory formation, may serve as a general mechanism to combat remaining pathogens. Given the overall low specificity of their innate and adaptive immune responses, it seems that turtles are able to adequately balance their immune compartments to eliminate pathogens, while simultaneously avoiding damage to self-tissues as a result of an overactive immune response.

### Gene family expansions

**Figure 6:**
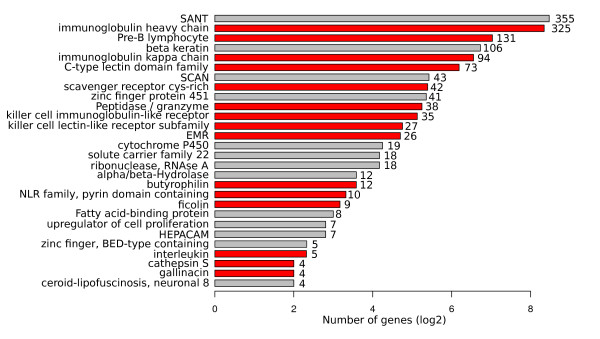
**Gene families showing expansion in the western painted turtle lineage**. The number of genes within a family is provided in front of each bar. Gene families associated with the immune response are shown in red.

Gene family expansions point to candidate sets of genes of particular importance in chelonian survival and evolution. After annotating the western painted turtle genome (see Materials and Methods, *Identification of gene family expansion/contraction*, Additional file 1, Table S16), we used phylogenetic reconstructions of the genomes of three mammals (human, mouse, platypus), two birds (chicken, zebrafinch), one lizard (green anole), and two fish (tetraodon, zebrafish) to identify one-to-one orthologs, as well as gene losses and gene family expansions in the western painted turtle genome. We identified 3,222 one-to-one orthologs across all nine species, 4,828 genes among the seven amniote species, and 103 gene families including 957 gene predictions that show expansion in the western painted turtle lineage. Among these expanded gene families, 15 of the 27 with four or more members, which jointly account for 623 of 957 gene predictions, were annotated as being involved in immune response (see Materials and Methods, *Expansion of gene families involved in the immune response*, Figure 6, Additional file 1, Table S17); an additional large expansion (106 members, 101 confirmed by manual curation) was evident among the beta-keratins (see Materials and Methods, *Beta-keratin expansions*) involved in the formation of scales, claws, and scutes that encase the shell [56]. Additional analyses using beta-keratin mRNAs extracted from the precursor cells of the shell of *Pseudemys nelsoni *[56] indicates that there have been independent lineage-specific expansions of the beta-keratins in birds and turtles associated with the formation of feathers and the shell (Li *et al*., unpublished results).

### Patterns of natural selection

Genomic scans for positive selection across turtles constitute a complementary strategy to identify genes underlying chelonian adaptations. We examined a carefully screened ortholog set of 4,136 genes (see Materials and Methods, *Ortholog sets*) for eight vertebrate species (human, platypus, chicken, zebrafinch, anole, turtle, python, and alligator) to detect signs of turtle lineage-specific positive selection. Using branch-site likelihood-ratio tests [57] with reduced parameterization [58] (see Materials and Methods, *Positive selection*), we identified 671 genes under positive selection (false discovery rate <0.1) (Accessory Data File 1). Among these genes were several categories of interest to notable physiological in turtles, several of which we highlight here.

There were nine genes containing ankyrin repeat motifs (the most significant was *ANKRD32*, *P *= 1.1x10^-17^), which are typically sites of protein-protein interactions. Furthermore, some of these ankyrin-repeat-motif genes contained SOCS box (suppressor of cytokine signaling) domains as well (*ASB14*, *P *= 1.2x10^-2 ^and *ASB18*, *P *= 8.8x10^-3^) and are involved in protein turnover regulation [59]. In addition, a number of chemokine receptors, *CCR4 *(*P *= 1.1x10^-7^), *CCR5 *(*P *= 7.2x10^-5^), and *CCR10 *(*P *= 1.0x10^-19^), as well as *CCRL1 *(*P *= 4.2x10^-9^), showed evidence of positive selection in our analysis. These G-protein coupled receptors bind specific cytokines (chemokines), are involved in chemokine-mediated signaling, and are generally pro-inflammatory/immune responsive [60]. We found evidence for significant positive selection in *DMRT2 *(*P *= 4.2x10^-4^). *DMRT*s have been found to associate with sexual determination and development (see earlier section on *Temperature-Dependent Sex Determination (TSD) genes*, also reviewed in [61]).

Related to oxidative phosphorylation and free-radical scavenging, several positively selected genes were involved directly (for example, *ATP5S*, *P *= 3.7x10^-6^; *ATP5H*, *P *= 3.1x10^-4^; *COX15*, *P *= 1.4x10^-4^; *ATP5G3*, *P *= 1.4 x10^-3^; *COX7A2*, *P *= 6.0x10^-3^; *ATP5B*, *P *= 1.9 x10^-2^; *DAP3*, *P *= 8.3x10^-6^) or indirectly (for example, *SOD1*, *P *= 1.5x10^-7^; *ACO2*, *P *= 1.4x10^-2^) in this process. Adaptations within genes in the process of ATP formation (specifically those that are subunits of ATP synthase) and anti-oxidant defenses have been proposed as mechanisms of life-history evolution in reptiles [62]. Several additional genes involved in life history traits were also under positive selection, including those involved in fertility (*FSHB*, *P *= 7.4x10^-6^), reproduction/immune functionality (*prolactin receptor*, *P *= 4.4x10^-8^), and aging (*SIRT3*, *P *= 2.1x10^-3^; *CLK1*, *P *= 1.1x10^-2^). In general, these 671 positively selected genes are involved in diverse functions that span biological processes. Although a numerically large set, our careful filtering and criteria for ortholog consideration suggests they are a robust set that is larger than would be expected when compared to naked mole rat or human [42,63].

We detected 171 GO functional categories showing enrichment for genes under positive selection (nominal *P *values <0.05, Mann-Whitney U-test), however, none were statistically significant after multiple testing correction (see Accessory Data File 1 for an overview of genes under positive selection and GO category enrichments).

## Conclusions

The western painted turtle, and chelonians generally, comprise a unique combination of extremely conservative evolutionary history interspersed with some of the most unique physiological and behavioral adaptations found in amniotes. Our analyses of the western painted turtle genome indicate that common vertebrate regulatory pathways are often involved with these novel phenotypes, and additional functional experiments can now investigate the ways in which these pathways have been modified in turtles. Our extensive analyses of anoxia tolerance provides particularly strong support for the interpretation that the western painted turtle utilizes common vertebrate pathways to achieve its extraordinary physiological abilities; temperature-dependent sex determination and immune system functionality also appear to utilize common suites of vertebrate genes. Genomic analyses of longevity and particularly tooth loss, both of which characterize all living chelonians, suggest that patterns of gene loss are also key elements of turtle evolutionary novelties. The western painted turtle genome, enabled by both comparative genomics and functional experimentation, has provided and will continue to provide windows into the evolution of physiological novelties, perhaps including some with biomedical and cryopreservation applications.

One aspect of turtle evolution that is proceeding at a rapid and accelerating pace is human-mediated extinction. Although the lineages represented by living turtles have survived countless challenges in the last 210 million years, current estimates are that at least 50% of the 330 recognized species of living chelonians are threatened with extinction [64]. Turtles far outstrip amphibians, mammals, and birds in their proportion of at-risk species, and the survival likelihood of many species is bleak. Future comparative genomics work on turtles, including comparisons among species that vary in their longevity, anoxia and freeze tolerances, immunocompetency, and a host of other key human challenges, requires healthy populations of the remaining diversity of turtles. The challenge, for comparative biology and conservation alike, is to preserve the remaining diversity of living turtles as we continue to unravel their secrets for success.

## Materials and methods

### Sequencing and assembly

A single *C. p. belli *(western painted turtle) was sequenced at The Genome Center, Washington University School of Medicine, St Louis, Missouri. The whole genome shotgun library primary donor-derived reads (B. Shaffer lab, female, field number: RCT428, locality: WA Grant Co, small lake 1.3 miles south of Potholes Reservoir, tissue accession number: HBS 112648) and BAC end reads (BAC library source: VMRC CHY3: J. Froula, JGI (from C. Amemiya lab) female, strain: MVZ #238119, Locality: Frenchman Hills wasteway 9.0 mi S via Dodson road of junction with Hwy I-90, Grant Co., Washington) were assembled using Roche's Newbler (version 2.6) with stringent parameters. Newbler uses all of the input single and paired end read data (including the paired BAC end data) to create contigs and then, focusing on the paired end read data along with estimates of insert size, organizes those contigs into larger scaffolds. After removing contamination, the resulting assembly was labeled as 3.0.1. All scaffolds >500 bases (81,642 scaffolds with a total size of 2.59 Gb, N50 scaffold size of 3.01 Mb (N50 number is 248)) were retained for submission to the public databases.

After the assembly was complete, 15X of paired end sequencing data were generated on the Illumina platform and used only for error correction in the reference assembly; the Illumina paired end data were not used to aid in scaffolding of existing contigs. For error correction, the Illumina data were aligned against the 3.0.1 assembly using bwa [65] and processed using samtools and bcftools [66]. Based on the paired end mapping data, all duplicate mapped reads were removed. One and two basepair indels were introduced into the reference for all cases where there were >3 and <200 reads aligned (mapping quality >40 and the indel was >10 bases from the end of the alignment), and where all reads disagreed with the reference and agreed with one another. There were a total of 27,296 indels introduced into 24,712 contigs.

The assembly data were aligned utilizing BLASTZ [67] to align and score non-repetitive turtle regions against the following repeat-masked genomes: anole (anoCar2), human (hg19), chicken (galGal3), and opossum (monDom5). Alignment chains differentiated between orthologous and paralogous alignments [68] and only 'reciprocal best' alignments were retained in the alignment set. The alignments were post-filtered in the following ways: (1) only alignments that extended over at least 2,000 bases where the relative expansion/contraction was <10X were retained; and (2) alignments were then smoothed by removing any single alignments that were <10 kb and occurred as a single alignment in between a large block of separate alignments to the same chromosome. The relative scaffold ordering was then examined in the four pairwise alignments. If at least three of the different pairwise alignments with the other species all suggested a given order and orientation, that pairwise ordering was retained in a list of valid orders (and orientations). Then the consistent pairwise alignments were linked into groups. The AGP was created using those lists of ordered and oriented scaffolds. Because ordering by homology is not absolutely confident, the gaps between scaffolds were annotated as 'contig' gaps including a 'no' in the final column indicating that there is no spanning clone closing the gap. There was approximately 1.2 Gb of sequence organized into 290 ordered groups leaving 80,697 individual scaffolds totaling 1.3 Gb. The N50 scaffold size rose to 5.2 Mb (N50 number is 148).

### Assembly quality and coverage assessments

As indicated by comparisons of the submitted assembly with a set of 64 finished western painted turtle BACs (BAC library source: VMRC CHY3; J. Froula JGI (from C. Amemiya, Benaroya Research Institute, Seattle, WA) Female; Strain MVZ #238119; Locality: WA: Grant Co: small lake 1.3 miles south of potholes reservoir) totaling 9.3 Mb of finished sequence, structural accuracy of the assembled sequence is sufficient for these analyses. These completed BAC sequences were not included in the assembly and thus provide an important dataset for assessing assembly accuracy and coverage. Some small supercontigs (most <5 kb) were not positioned within larger supercontigs (<1 event per 500 kb). While these are not strictly errors, they do affect overall assembly statistics. There are also small, undetected overlaps (most <1 kb) between consecutive contigs (approximately 1 event per 30 kb), occasional local mis-ordering of small contigs (approximately 1 event per Mb), and small contigs incorrectly inserted within larger supercontigs (<1 event per 275 kb). Overall, the rate of rearrangements with respect to finished BACs was comparable to previous next generation WGS assemblies. Nucleotide-level accuracy is high by several measures. Over 99% of the consensus bases in the western painted turtle sequence have quality scores [69] of at least Q40 corresponding to an error rate of ≤10^-4^. Comparison of the WGS sequence to the 9.3 Mb of finished BACs from the sequenced individual is consistent with this estimate, giving a high quality discrepancy rate of 3x10^-3 ^substitutions and 2x10^-4 ^indels which is no more than expected given the heterozygosity rate. The rate of substitutions is due to the polymorphism rate. By restricting analysis to high-quality bases, the nucleotide-level accuracy of the WGS assembly is sufficient for analyses presented here. As with the chimpanzee and other whole genome shotgun-based assemblies, the most problematic regions are those containing segmental duplications (Chimpanzee Sequencing and Analysis Consortium, 2005).

We estimate that western painted turtle genome sequence covers at least 93% of the full genome sequence. To obtain this estimate, we first evaluated the coverage using the results of the alignments of the assembly against the 64 finished western painted turtle BACs. The overall coverage of those BACs exceeded 93%. Second, we aligned a set of western painted turtle cDNAs generated by this project against the genome assembly using BLAT [70]. The cDNA libraries were constructed from several tissue sources (see Additional file 1, Table S2) and were sequenced in our lab on the 454 Life Sciences instrument using methods previously reported [71]. The reads were assembled using the Newbler software package provided by 454 Life Sciences. The coverage estimates per tissue range from 93% to 98% when asking that at least 50% of the EST align to the genome or from 91% to 96% when requiring more than 90% of the EST aligns to the genome (see Additional file 1, Table S2).

Finally, we estimated coverage by looking at the coverage of a related genome using BLAT [70]. Over 96% of the draft assembly of the 1.5 Gb *Trachemys scripta *genome (separated by approximately 10-15 My from the western painted turtle) aligned with the western painted turtle genome.

### Repeat structure

TE sequence divergence in three turtle genome assemblies reveal a distribution that contrasts with the high turnover of younger L1s in the lizard (*Anolis*), the skewed accumulation of older TEs in the alligator, and near complete lack of SINEs and active CR1s in the small, homogenous genomes of birds (see Additional file 1, Figures S1-S5) [9,11,72]. The average G+C content of *C. p. bellii *mobile elements is the same as the genome-wide average of 43% and the range of values for TE content and G+C among the N50 scaffolds is more similar to those observed in chicken than in *Anolis *(see Additional file 2, Figure S6) [9], consistent with its closer phylogenetic relationships to archosaurs.

Identification and classification of repetitive elements in the *C. p. bellii *assembly were carried out on the full original *C. p. bellii *assembly sequence (*C. picta bellii *v3.0.1) using the RepeatMasker version 3.3.0 [8], Tandem Repeat Finder version 4.0.4 [73], and Phobos version 3.3.12 [74] software packages. For all available genome assemblies investigated RepeatMasker was run with the BLAST engine and repeat classification was carried out using the Vertebrate library from version 20110920 of the RepBase database. We employed Phobos using default parameters. Tandem Repeat Finder was run with the default alignment parameters except for a reduced MaxPeriod value of 200 instead of the default 500, and with exclusion of HTML output. These parameter settings were directly comparable to summary statistics available through TRDB for the most recent whole-genome assemblies of amniote species. Results from RepeatMasker were analyzed using RMPipeline [75], a set of generalized programs for analyzing RepeatMasker output written using Perl. These programs can be used to process any RepeatMasker output files and are publicly available and free to use under the GPLv3 license. Graphs were created using RMPipeline results, some additional Perl scripts, and Microsoft Excel.

### Isochores

The absolute GC content of the assembly (after removing scaffolds with >20% missing data) is 0.434. We examined whether the assembly exhibited any bias in GC content. We divided the assembly into four equally-sized bins of increasing scaffold size (after omitting scaffolds with >20% missing data). The absolute GC contents of each bin were (range of scaffold lengths are indicated in bp): 0.496 (501-591), 0.498 (591-735), 0.496 (735-1,039), 0.433 (1,039-26,452,378). Because it appears there is a bias for smaller scaffolds to have a larger GC proportion, we focused our analyses of genomic GC content to those >320 kb, a subset of the genome whose absolute GC is 0.430, a value very close to the whole-genome absolute GC. To generate the distributions of GC content, we divided up the genomes of human, dog, frog, turkey, zebrafinch, chicken, and western painted turtle (scaffolds >320 kb) into 3-kb windows, using the GC content of these windows as measures (see Additional file 2, Figure S7). We also examined GC variation at different spatial scales, using non-overlapping windows of 5, 20, 80, and 320 kb (Figure 1). As window size quadruples, standard deviation should decrease by 50% for a completely homogeneous genome [76]. To determine the relationship between GC3 and flanking sequence, we used 10 kb upstream of the start codon and 10 kb downstream of the stop codon as the 20-kb flanking sequence. Only those flanking sequence with 80% complete data (allowing 20% combined missing data or clipped ends due to proximity of the gene to the ends of the scaffold) were considered. To examine the relationship between gene density and GC content, we divided up intergenic sequences into 10 equal-sized bins of increasing size and calculated the GC content of each bin. For the western painted turtle, we found a weak but significant correlation between the GC content of protein-coding genes and their flanking sequence, indicating that genomic environment influences the nucleotide composition of genes (see Additional file 2, Figure S8).

### Multiple alignments and gene orthologs

Comparative genomic analyses (including studies of phylogenetic relationships, selection, conserved elements, and accelerated regions) are prone to artifacts derived from biases introduced by differences in gene prediction methods used in draft genome annotations of individual genomes included in the study, as well as gene prediction errors. In order to avoid having such biases dominate analyses, one can chose a well-annotated reference genome (in our case, human or chicken, whichever is more appropriate for a particular analysis), and annotations are remapped from the reference to the target genomes through multiple alignment. This step is followed by extensive checks to ensure the quality of derived annotations in target genomes.

A disadvantage of this approach is that novel elements introduced in non-reference genomes are not covered by the analysis. In case of human-referenced orthologs, the analysis only includes genes preserved throughout amniote evolution (since mammals are the sister groups of the remaining amniotes), while in the case of chicken-referenced orthologs, we analyze genome elements preserved during the evolutionary diversification of turtles and archosaurs (see Figure 2). Thus, reference derived ortholog sets are best used in analyses requiring conservative high-confidence gene sets, and are not suitable for estimating target genome characteristics, such as numbers of genes, exons, or novel elements.

To construct a set of high-confidence orthologs, we used a methodology developed by Kosiol and colleagues [58]. First, we created a multiple alignment of human (hg19), platypus (ornAna1), chicken (galGal3), zebrafinch (ornAna1), anole (anoCar2), turtle, python, and alligator, using a standard UCSC genome browser pipeline [77] based on BLASTZ [67] and multiz [78]. We based ortholog predictions on the human gene catalog of 21,360 genes (including RefSeq, UCSC known genes, ENSEMBL, and VEGA genes), which were remapped to all of the above species through these multiple alignments. We observed high variability for positions of translation start sites and stop codons, thus we also evaluated incomplete gene models, where we removed 10% on each end of the gene. Altogether, our gene set contained more than 378,000 alternative gene models.

Series of filters were run to identify which of these gene models can be considered high-confidence orthologs. For a gene model to be considered clean in a particular genome, we required that: (1) it was covered by a single chain within the syntenic (for platypus and chicken) or reciprocal-best (for zebrafinch, anole, turtle, python, and alligator) net created using the UCSC genome browser pipeline; (2) there were no significant gaps in the gene alignments; (3) there were no frameshifts uncorrected within a short window of sequence; and (4) all elements important for the gene structure (donor sites, acceptor sites, translations start sites, and stop codons) were preserved. For each gene, we selected a single gene model that was clean in turtle, giving preference to the models that were clean in the most species and were the longest. The gene was excluded if it did not have any gene model satisfying these conditions (see Additional file 1, Table S6, which shows the number of genes filtered out in each step.) This approach resulted in 4,786 high-confidence orthologs, out of which 3,318 are incomplete (shifted start codon or stop codon). Out of these genes, 312 covered two species (human and turtle), 622 covered three species, 757 covered four species, 896 covered five species, 1,048 covered six species, 842 covered seven species, and 309 covered all eight species. An additional 12 genes that were incompletely covered in the reference genome were detected in the last stages of comparison and removed in postprocessing.

### Phylogeny and substitution rate

We estimated phylogeny using the set of 1,955 orthologs that we identified in at least five of the eight genomes that we examined and that had the potential to be informative about the phylogenetic position of turtles. We partitioned the dataset by codon position, using an independent GTR model for each position and allowing for gamma-distributed rate variation among sites. We ran four independent analyses for 10 million generations, sampling every 1,000 generations in MrBayes v. 3.1.2 [79]. We then estimated the relative rate of substitution in a smaller dataset that was designed to minimize missing data. This dataset comprised 309 orthologs that were identified in all eight species. We used a UCLN relaxed clock model implemented in BEAST v. 1.7.1 [80]. We partitioned the dataset by codon position, using independent general time reversible models of DNA substitution allowing gamma distributed rate variation for each position. We set the log normal distribution describing among-branch substitution rate variation to mean 1.0 and standard deviation of 0.33 and estimated relative substitution rates on the topology shown in Figure 2. We carried out three replicate runs, ensuring convergence and adequate mixing by inspecting samples from the MCMC in Tracer [81]. Each analysis was run for 10 million generations and sampled every 1,000 generations. Rates varied by a factor of approximately 5, ranging from the lowest relative rate of 0.33 (in turtle) to a high of 1.67 (in python; see Additional file 1, Table S5).

### Anoxic gene expression

To better understand the transcriptomic changes that might underlie the profound anoxia tolerance of the western painted turtle, differential gene expression was investigated in telencephalon and ventricle from western painted turtles that were either normoxic or submerged in anoxic water 24 h at 19°C (*n *= 4 per group, 8 total; mean ± SD 238.6 ± 23 g, range, 198-274 g) using RNA-seq methodology. At the end of the submergence period, the turtles, which appeared sedated due to profound metabolic depression, were removed from the chamber and quickly euthanized. The telencephalon was removed from the braincase, stripped of any adherent meninges, and flash-frozen in freeze-clamps previously cooled in liquid nitrogen. A 2 × 4 cm window was quickly cut in the plastron with a bone saw, exposing the still-beating heart, which was quickly removed, bisected, blotted on gauze to remove any blood, and quickly flash-frozen. Water was considered anoxic when oxygen concentrations were undetectable with a submerged oxygen electrode (YSI D200) while bubbling the water with nitrogen gas. Frozen tissue samples (22-109 mg) were ground to a fine powder under liquid nitrogen with a mortar and pestle and transferred to a dry-ice cooled test tube with a liquid nitrogen-cooled spatula. One milliliter of room-temperature Trizol^® ^reagent (Life Technologies) per 50-100 mg tissue was added to the tube, which was immediately vortexed. All subsequent RNA isolation steps were performed according to the Trizol manufacturer's instructions. The final RNA pellet was resuspended in DEPC-treated water and treated with DNAse I (Life Technologies) according the manufacturer's instructions in order to remove any DNA contamination. RIN values for the samples were all >7.4 (Agilent 2100 Bioanalyzer). cDNA library construction and sequencing was carried out using previously described method [11,82].

Paired-end 2x100 bp reads generated from poly(A) selected RNA-seq libraries from all 16 samples were aligned to the latest *C. p. bellii *assembled reference sequence, using TopHat 1.4.0 [83], which also splits reads to align them across known and novel splice junctions. For known splice junction loci, a GTF (Gene Transfer Format) file of OPTIC annotations was provided. To estimate transcript and gene abundances, Cufflinks 1.3.0 [84] was used. This generates normalized FPKMs (Fragments per kilobase of exon model per million mapped fragments) for each annotated gene and transcript as defined in the OPTIC based annotations. The Cufflinks parameter -G was used to exclude novel isoforms, in order to exclude large outliers (regions with extraordinarily high read-depths) that causes the Cufflinks normalization method to introduce a loss of sensitivity. The per-gene FPKMs were log_2 _transformed and compared across treatments and tissues by ANOVA assuming a normal/Gaussian distribution [85] with FPR multiple testing correction using JMP Genomics 5.1. Genes were excluded from the analysis if the median FPKM equaled zero for three out of the four sampling groups. The results of genes showing greater than two-fold increases are shown in Additional file 1, Tables S7, S8; down-regulated genes are shown in Additional file 1, Table S9; and RNA-seq read depths for the most highly up and down-regulated genes are shown in Additional file 2, Figures S10-S12.

### Freeze tolerance

The Mfold (v.2.3) computer program was used to predict RNA structure [86]. The program predicts secondary structure based on the energy minimization method and thermodynamic parameters. We initially searched the *C. p. bellii *assembly (v.3.0.1) for the sequence of premiR-29b using BLAST+ (v.2.2.18) [87]. We focus on this micro-RNA because, in conjunction with ongoing experiments (Storey, unpublished results), we found that miR-29b increases in expression levels for many models of metabolic rate depression (hibernating mammals, freeze tolerance, and anoxia tolerance). This is most likely due to its proposed role in regulating the PI3K/Akt signaling pathway, a pathway that is commonly differentially regulated in response to environmental stress and has been shown to control glucose metabolism and transport, survival (apoptosis), translation processes and cell cycle arrest. This microRNA continually proves to be a utilized regulatory response to severe environmental stresses. Small RNAs, including miRNAs, were isolated using the mirVana miRNA isolation kit from Ambion Inc. (P/N: 1560) according to the manufacturer's protocol. Samples (approximately 100 mg) were homogenized 1:10 w:v in lysis/binding buffer, left on ice for 10 min and then a mixture of acid phenol:chloroform was added in a 1:1 ratio. Samples were centrifuged for 5 min at 10,000 ×g and the supernatant was collected. Small miRNAs (<200 nt) were isolated using the enrichment protocol provided with the kit involving two sequential filtrations through glass-fiber filters at different ethanol concentrations. RNA concentration was determined by absorbance at 260 nm and the ratio of absorbance at 260/280 nm was used as an indicator of RNA purity.

To determine the expression of mature miR-29b from *C. p. bellii*, a modified v miRNA-specific reverse transcription and qRT-PCR procedure was performed. A 5.0 µL aliquot of small RNA (0.2 ng/µL) was incubated with 1µL of 250 nM microRNA-specific stem-loop primer (5'-CTCACAGTACGTTGGTAT CCTTGTGATGTTCGATGCCATATTGTACTGTGAGAACACTGA-3'). The reaction was heated at 95°C for 5 min to denature the RNA, and then incubated for 5 min at 60°C to anneal the stem loop primer. After cooling on ice for 1 min, the remaining reagents (4 µL of 5x first strand buffer, 2 µL of 0.1 M DTT, 1 µL of dNTP mixture containing 25 mM of each nucleotide, and 1 µL of M-MLV reverse transcriptase) were added. The reaction proceeded for 30 min at 16°C, followed by 30 min at 42°C, and 85°C for 5 min. Following reverse transcription, the RT product was stored at -20°C. Real-time PCR was performed on a BioRad MyiQ2 Detection System (P/N: 170-9790, BioRad). The 25 µL qRT-PCR reaction included 5 µL RT product, 12.5 µL SsoFast EvaGreen Supermix (P/N: 172-5201, BioRad), 0.5 µL of 12.5 µM forward primer (5'-ACACTCCAGCTGGGTAGCACCATTTGAAATC-3'), 0.5 µL of 12.5 µM reverse primer (5'-CTCACAGTACGTTGGTATCCTTGTG-3'), and 6.5 µL nuclease free water. Reactions were incubated in a 96-well plate at 95°C for 3 min, followed by 40 cycles of 95°C for 15 s and 60°C for 1 min. A melting curve analysis was performed for each miRNA analyzed. All reactions were run in triplicate.

### Tooth loss

We initially searched the *C. p. bellii *scaffolds for individual exons of *ENAM*, *AMEL*, *AMBN*, *DSPP*, and *MMP20 *using BLAST+ (v. 2.2.18) [87]. We used crocodilian sequences for *AMEL *- AF095568, *AMBN *- AY043290, and *ENAM *- GU344683.1, MMP20 - DQ885891.1 and human sequences for *DSPP *- NM_014208 and part of *MMP20*- NM_004771.3. We were able to identify from one to several conserved exons from the *C. p. bellii *pseudogenes, thus providing us with an anchor point for further analysis. Subsequently, we utilized UniDPlot, which is a tool for the detection of poorly conserved DNA regions and was previously used to find pseudogenes in chicken [41,88]. Finally, we utilized T-coffee to align homologous exons and manual curation to identify GT-AG exon-intron junctions. Identity scores were calculated using LALIGN [89]. Gene positions within chromosomally syntenic regions were analyzed using lizard (*A. carolinensis *genome assembly 2.0), chicken (*Gallus gallus *genome assembly 4.0), and the UCSC Human Genome Browser.

### Aging and longevity

We obtained the individual exon sequences for all five shelterin complex encoding genes and for the genes in HAGR, from NCBI. We then searched all *C. p. bellii *sequence and RNA-seq data to identify orthologs of individual exons of these genes using BLAST+ (v. 2.2.18) [87]. We used either chicken or anole (lizard) sequences as the query sequence. While *TEP1*, *TERF2IP*, and *ATP5O *were completely absent from *C. p. bellii *genome, partial fragmented forms of the other genes were found. To avoid draft assembly artifacts, we confirmed our results by carrying out similar searches for these genes in all four turtle genome available to us (See Additional file 1, Table S12).

### Sex determination/differentiation

454 reads (generated by us) from the transcriptomes of *C. p. bellii*, *Chelydra serpentina*, *Apalone mutica*, and *Podocnemis expansa *were combined and mapped to *C. p. bellii *assembly 3.0.1 using GMAP [90]. The resulting SAM file was then run through Cufflinks 1.3.0 [91] to obtain a GTF file containing a single list of putative genes in the western painted turtle genome. This GTF file was used in CuffLinks as the reference GTF for subsequent CuffLinks runs on each 454 dataset. cDNA sequences per tissue and species were extracted using R and the bioconductor package ShortRead from this reference GTF file. Each transcriptome 454 dataset was mapped to the *C. p. bellii *assembly 3.0.1 using GMAP.

DNA coding sequences from the genomes or transcriptomes of multiple vertebrates of 34 genes in the sex determination/differentiation network of vertebrates or linked to sex chromosomes in chicken (see Additional file 1, Table S13) were extracted and aligned using CLUSTALW in Geneious Pro [92] and artificial frameshifts and other errors were manually corrected. Rates of molecular evolution were evaluated by calculating dN, dS, and dN/dS per gene in MEGA5 [93]. Tests of neutrality, positive and purifying selection were carried out in MEGA5 using the codon-based Z-test, using the Nei-Gojobori method [94], where the variance of the difference was computed using the bootstrap method with 500 replicates. Optimal models of DNA evolution were inferred per gene and gene-specific phylogenetic trees were built by maximum likelihood with MEGA5, and topologies contrasted among genes with the species phylogenetic relationships.

### Immune system

A unique sequence database was generated from Ensembl [95] consisting of approximately 3,000 immune genes from human (*Homo sapiens*, GRCh37), mouse (*Mus musculus*, NCBIM37), rat (*Rattus norvegicus*, RGSC3.4), chicken (*Gallus gallus*, WASHUC2), Fugu (*Takifugu rubripes*, FUGU4), Medaka (*Oryzias latipes*, MEDAKA1), Anole (*Anolis carolinensis*, AnoCar2.0), Stickleback (*Gasterosteus aculeatus*, BROADS1), Turkey (*Meleagris gallopavo*, UMD2), Xenopus (*Xenopus tropicalis*, JGI_4.2), Tetraodon (*Tetraodon nigroviridis*, TETRAODON8), Zebrafinch (*Taeniopygia guttata*, taeGut3.2.4), Zebrafish (*Danio rerio*, Zv9), and Sea Lamprey (*Petromyzon marinus*, Pmarinus_7.0). Sequences, Ensembl Gene ID, and Gene Name were obtained from Ensembl directly or using the Biomart mining utility [96] when available. Pairwise alignments were obtained using in-house BLAST (BLASTN 2.2.15) [97] comparing query immune gene sequences to the *C. p. bellii *genome assembly and unassembled sequencing reads, gene predictions, and cDNA reads.

### Identification of gene family expansion/contraction

To identify gene family expansions and contractions, we built phylogenetic trees for all predicted genes in *C. p. bellii *with their orthologs in human, mouse, platypus, chicken, zebrafinch, green anole and using the pufferfish and zebrafish as outgroups.

Orthology assignments and orthologous groups were defined using the OPTIC pipeline [10,98]. Orthology assignments are based upon the computation of pairwise orthologs using PhyOP [99] using BLASTP searches with an E-value threshold of 10^-5 ^and a minimum size cut-off equal to 75% of the smaller sequence. The alignments were weighted according to the normalized bit score:

$${\text{s}}_{\text{ij}}\text{ = 1 - ((max[s'ij,s'ji])/min(s'ij,sji)class="textsf" mathvariant="sans-serif">.}$$

Where s'_ij _is the bit score for a BLASTP alignment between sequence i and j.

A tree-based orthology method implemented within PhyOP [99] was used to define clusters of orthologous groups. For each cluster, genes were aligned using MUSCLE [100], genes with multiple transcripts were collapsed into sequences of non-redundant exons and phylogenetic trees were estimated using TreeBeST [101]. Rates of non-synonymous substitutions per non-synonymous sites (*d_N_*) and rates of synonymous substitutions per synonymous sites (*d_S_*) and their ratio (*d_N_*/*d_S_*) were estimated for each branch of the tree with PAML [102]. Rates were not allowed to vary between sites. To remove biases associated with poor alignments, translated sequences were masked with SEG [103] and corresponding masked codons were removed; poorly aligned columns were also removed using Gblocks [104].

A total of 20,234 orthologous groups were found, of which 12,938 have at least one gene prediction from *C. p. bellii *and 1,176 groups contain at least two *C. p. bellii *gene models. All orthology/paralogy predictions are available at [105]. We identified a total of 4,828 genes with one-to-one orthologous relationship between all amniotes, and 3,222 between all species when pufferfish and zebrafish are included. A total of 604 predicted gene models in *C. p. bellii *had no predicted orthologs; these include rapidly-evolving genes as well as problematic gene models that survived our filters. We also identified 568 groups with genes in human, mouse, platypus, chicken, zebrafinch, and green anole but that have no detectable orthologs in the current version of the *C. p. bellii *genome assembly. These currently absent genes will contain genes absent from the current assembly, as well as rapidly-evolving genes.

In order to reach a conservative estimate of the number of genes within a family and to remove any residual biases associated with the assembly process, we estimated the pairwise amino acid identity between every pair of members of a family and rejected duplicated genes that are more than 97% identical. A summary of gene expansions is presented in Figure 6.

### Beta-keratin expansions

Beta keratins have previously been described to be an important component of the corneous layers of the reptilian epidermis forming the scales, claws, and beak. In birds, they are the major component of feathers [106]. We identified a total of 106 gene models (101 complete) in *C. p. bellii *that share significant sequence similarity with avian and green anole beta-keratins. Using beta-keratin mRNAs extracted from the precursor cells of the shell of *Pseudemys nelsoni *[56], and the phylogeny built with PhyML [107], we identified 41 and 60 putative non-shell and shell proteins in *C. p. bellii*, respectively.

### Expansion of gene families involved in the immune response

Among the families with the largest expansions (≥4 members), 15 are related to the innate or adaptive immune response.

As part of the adaptive immune response, we identified 365, 131, and 94 predicted gene models in *C. p. bellii *that cluster with the immunoglobulin heavy chain, lambda, and kappa chain variable regions respectively in mouse. The large number of genes from these two families is of prime importance in the generation of antibody diversity through V(D)J recombination. Both the immunoglobulin heavy and light chain variable regions are known to be among the most dynamic gene regions in the human genome, and immunoglobulin genes are known to show high allelic and copy number variation [108,109]. Interestingly the imunoglobulin kappa chains have been lost in the bird genomes [110]. These authors predicted this loss to predate the divergence between Passeriformes and Galliformes (100 Mya). In agreement with this, our analysis shows that the immunoglobulin kappa chains were present in the common ancestor of the birds and turtles approximately 260 million years ago.

We also identified expansions of several gene families that form part of the innate immune system. These gene products are expressed on the surface of natural killer (NK) cells (NK cells' C-type lectin-like and NK cells' immunoglobulin-like receptors) or are secreted by these NK cells (for example, granzymes). NK receptors previously shown to belong to the LCR in human, mouse and chicken are known to have undergone lineage-specific expansion in each of these lineages [111-114]. We searched the *C. p. bellii *polypeptide predictions belonging to these two families for transmembrane domains [115] and found that only six of 27 putative NK cells' C-type lectin-like and 14 of 35 putative NK cells' immunoglobulin-like receptors possess transmembrane domains.

### Ortholog sets

We based our study of positive selection on the set of carefully screened orthologs for eight vertebrate species (human, platypus, chicken, zebrafinch, anole, turtle, python, and alligator; see Materials and Methods, *Multiple Alignments and Gene Orthologs*). From among 4,786 high-confidence ortholog sets, each covering between two and eight species, we selected 4,136 sets that covered human, turtle, and at least one of the outgroup genomes (chicken, alligator, zebrafinch).

### Positive selection

We detected signs of positive selection using likelihood ratio tests [57] with reduced parameterization [58]. *P *values were estimated assuming a null distribution that is a 50:50 mixture of χ*2 *distribution with one degree of freedom, and a point mass at zero, leading to conservative *P *value estimates [116]. The branch leading to the turtle was designated as a forward branch, with some sites allowing dN/dS>1, while all other branches were background branches, disallowing positive selection. The results were corrected for multiple testing using Benjamini and Hochberg false discovery rate control (FDR). Accessory Data File 1 shows the results of likelihood-ratio tests for all genes with nominal *P *values <0.05 (890 genes), indicating genes with FDR <0.1 (671 genes).

We also examined GO functional categories for enrichment for genes under positive selection, using Mann-Whitney U-test with Holm's correction for multiple testing [117]. No functional categories were statistically significantly enriched for genes under positive selection after multiple testing correction. Accessory Data File 1 shows 171 GO categories with nominal *P *values <0.05.

## Competing interests

The authors declare that they have no competing interests.

## Authors' contributions

HBS, PM, AMS, RCT, and NV comprise the organizing committee of the western painted turtle genome sequencing project. Manuscript organization and editing: HBS, PQS, RCT, WCW, CPP, WH, and PM. BAC library construction: CTA. Project management and data production: LF, KDD, CCF, MO, and TAG. Assembly and analysis: PM, NT, and LWH. RNA-seq and anoxia analysis: DEW, CK, and LTB. Freeze tolerance: KBS and KKB. Gene model predictions, orthology prediction, and analysis: CPP, WH, LK, and YL. Repeat element and isochore analysis: AMS, CWB, and MKF. Comparative alignments: BJR. Phylogenetic analyses: RCT, TV, TAC, DDP, APJK, REG, JSt.J, and ELB. Gene analyses, sex chromosome genes: FJJ, SEM, AMB, TS, AS, NV, RL, DB, SR, DJ, SVE, BC, MC, OH, and LM. Gene analyses, aging: RH. Gene analyses, immuno-response: RMB, GMB, LMZ, and RTP. Gene analyses, enamel: JA and JMR. Turtle accelerated regions: AKH and BGB. Principal investigators: ERM, WCW, and RKW. All authors read and approved the final manuscript.

## Author information

The *Chrysemys picta bellii *whole-genome shotgun project has been deposited in NCBI GenBank under the project accession AHGY00000000. The raw input data for *Chrysemys picta bellii *(BioProject ID: 78657) was deposited to the trace archive and the SRA under the project accession SRP012057. The *Apalone spinifera *whole genome data can be found at the NCBI SRA under the accession numbers SRX217616-7. Specimen collection for the *C. picta bellii *was authorized by the Washington Department of Fish and Wildlife under scientific collecting permit 08-086 (to RCT) and complied with IACUC standards at UC Davis (HBS protocol holder). Ethical (IACUC) approvals for all experiments involving living turtles were obtained at the university where the experiment or field work were conducted.

## Description of additional files

We provide three Additional Files. Two Additional Files are in Microsoft word (.docx). Additional file 1 contains Tables S1-S17), and Additional file 2 contains Figures S1-S12). We also provide Additional file 3 in support of the selection scan analysis in Microsoft Excel format. In it, the sheet labeled **mwu_turtle_go **details enrichment of GO categories for positive selection on the western painted turtle lineage with nominal *P *values <0.05 (Mann-Whitney U-test). No categories were significantly enriched for positive selection after application of multiple testing correction. The second sheet, labeled **lrt_turtle**, shows genes under positive selection on the western painted turtle lineage. All genes with nominal *P *values <0.05 (likelihood ratio branch-site test) are shown, and the 671 genes that were statistically significant after applying multiple testing correction (FDR <0.1) are also noted.

## Supplementary Material

Additional file 1 **Supplementary tables**. Tables S1-S17 contain additional information in support of the painted turtle assembly (Tables S1-S2), transposable elements (Table S3), isochores (Table S4), phylogeny and evolutionary rates (Tables S5-S6), anoxia (Tables S7-S9), tooth loss (Tables S10-S11), longevity (Table S12), sex determination (Table S13), immune function (Tables S14-S15), and gene family expansions (Tables S16-S17). Click here for file

Additional file 2 **Supplementary figures**. Figures S1-S12 contain additional information in support of the painted turtle repeat analyses (Figures S1-S6), isochores (Figures S7-S9), and anoxia tolerance (Figures S10-12). Click here for file

Additional file 3 **Selection scan analysis**. The sheet labeled **mwu_turtle_go **provides additional information on the enrichment of GO categories for positive selection on the western painted turtle, while the sheet labeled **lrt_turtle **lists all genes under positive selection on the western painted turtle lineage. All genes with nominal *P *values <0.05 (likelihood ratio branch-site test) are shown, and the 671 genes that were statistically significant after applying multiple testing correction (FDR <0.1) are also noted. Click here for file
