# Supplementary material for: The western painted turtle genome, a model for the evolution of extreme physiological adaptations in a slowly evolving lineage
Source: Genome Biol. 2013 Mar 28;14(3):R28. doi: 10.1186/gb-2013-14-3-r28 (PMC4054807; doi:10.1186/gb-2013-14-3-r28)
Supplement: Additional file 1 — Supplementary tables. Tables S1-S17 contain additional information in support of the painted turtle assembly (Tables S1-S2), transposable elements (Table S3), isochores (Table S4), phylogeny and evolutionary rates (Tables S5-S6), anoxia (Tables S7-S9), tooth loss (Tables S10-S11), longevity (Table S12), sex determination (Table S13), immune function (Tables S14-S15), and gene family expansions (Tables S16-S17). [file gb-2013-14-3-r28-S1.DOC]

**Supplementary Tables**

**Table S1.** Reads used as input to the Western Painted Turtle whole genome shotgun assembly

| Read | Insert |  | > phred 30 | Sequence | Physical |
| --- | --- | --- | --- | --- | --- |
| Type | Size (kb) | Reads (M) | Bases (M) | Coverage | Coverage |
| 454_frag | 0.5 | 119.3 | 26645 | 10.3 | 23.0 |
| 3kbFLX | 3.0 | 6.8 | 1513 | 0.6 | 3.9 |
| 3kbNick | 3.0 | 85.0 | 18352 | 7.1 | 49.2 |
| 8kbPE | 8.0 | 22.3 | 51130 | 1.9 | 34.5 |
| BES | 145.0 | 0.3 | 135 | 0.1 | 8.0 |
| Total |  | 233.7 | 51758 | 19.9 | 118.6 |

*based on reads submitted to the assembler

**Table S2** Percentage of Western Painted Turtle ESTs aligned to the genome assembly by tissue

|  | input | contigs | aligned over this % of EST length | | | |
| --- | --- | --- | --- | --- | --- | --- |
| tissue | reads | >500 bases | >=9% | >=20% | >=50% | >=90% |
| brain | 412899 | 4014 | 99.6 | 98.7 | 98.3 | 96.2 |
| ovaries | 417089 | 2200 | 95.4 | 94.3 | 93.6 | 91.2 |
| testes | 491219 | 3368 | 97.8 | 96.9 | 96.5 | 94.2 |
| trunks | 494188 | 4783 | 99.8 | 98.7 | 98.3 | 95.9 |
| total | 1815395 | 11351 | 98.2 | 97.1 | 96.7 | 94.3 |

**Table S3.** Summary of Transposable Elements in the *C. picta* assembly based on RepeatMasker annotation.


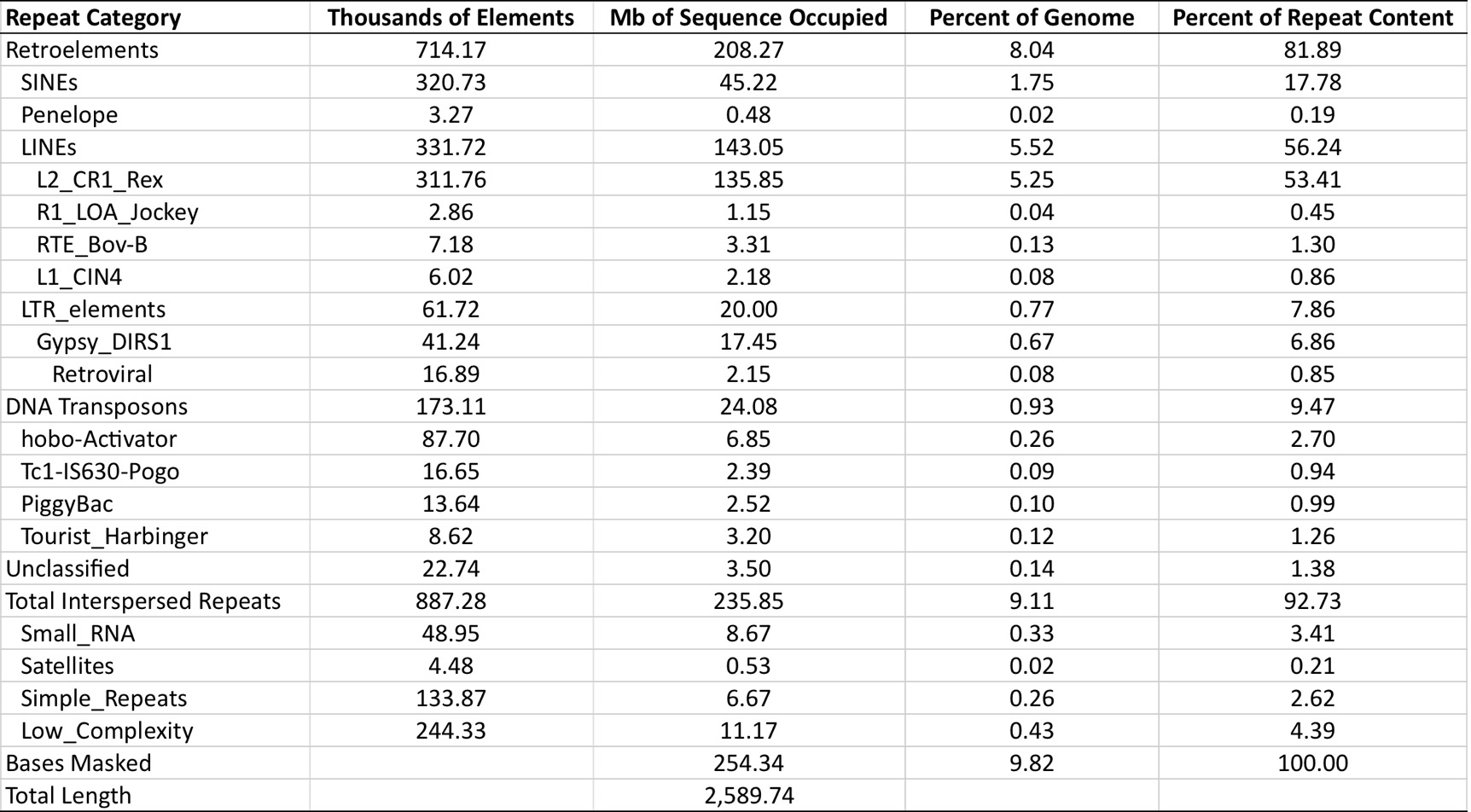


**Table S4.** Kolmogorov-Smirnov tests of d*N*/d*S* distributions between low-GC3 and high-GC3 genes in human, chicken, and Western Painted Turtle.

|  | **median dN/dS** | | | **mean dN/dS** | | |  |
| --- | --- | --- | --- | --- | --- | --- | --- |
|  | **low GC3** | **high GC3** | **difference** | **low GC3** | **high GC3** | **difference** | **KS p-value** |
| **Human** | 0.065 | 0.046 | 0.019 | 0.131 | 0.139 | -0.008 | 1.14E-09 |
| **Chicken** | 0.075 | 0.046 | 0.029 | 0.110 | 0.095 | 0.015 | 6.47E-11 |
| **turtle** | 0.116 | 0.068 | 0.048 | 0.175 | 0.143 | 0.032 | 2.20E-16 |

**Table S5.** Posterior relative rate estimates from UCLN relaxed clock analysis.

|  | platypus | chicken | zebrafinch | lizard | turtle | python | alligator |
| --- | --- | --- | --- | --- | --- | --- | --- |
| mean | 1.1114 | 0.6834 | 0.8315 | 1.4125 | 0.3387 | 1.6715 | 0.4545 |
| 95% HPD lower | 1.1105 | 0.6745 | 0.8247 | 1.4056 | 0.3282 | 1.6565 | 0.4439 |
| 95% HPD upper | 1.1123 | 0.6918 | 0.8378 | 1.4199 | 0.3484 | 1.6873 | 0.4645 |

**Table S6.** The number of genes left after application of each of the filters. These numbers reflect both evolutionary distance from human genome, and the quality of the assembly

|  | platypus | chicken | zebrafinch | lizard | turtle | python | alligator |
| --- | --- | --- | --- | --- | --- | --- | --- |
| All genes | 21360 | 21360 | 21360 | 21360 | 21360 | 21360 | 21360 |
| (a) synteny | 8597 | 7497 | 6509 | 7601 | 8127 | 2300 | 7764 |
| (b) gaps | 7933 | 7423 | 6454 | 7541 | 7632 | 2136 | 7234 |
| (c) frameshifts | 6138 | 5779 | 4911 | 5719 | 5244 | 1425 | 5048 |
| (d) gene structure | 5652 | 5274 | 4427 | 5128 | 4786 | 1328 | 4557 |

| **Table S7.** List of transcripts which increased in Western Painted Turtle telencephalon after 24 hours of anoxia at 19°C. Data are from analyses involving Log-Normal multiple comparisons model. | | |
| --- | --- | --- |
| **Human Gene**  **Ortholog** | **Gene**  **Description** | **Fold**  **Change** |
| APOLD1 | apolipoprotein L domain containing 1 | 128.8 |
| PTGS2 | prostaglandin-endoperoxide synthase 2 (prostaglandin G/H synthase and cyclooxygenase) | 28.8 |
| FOSB | FBJ murine osteosarcoma viral oncogene homolog B | 24.7 |
| FOS | FBJ murine osteosarcoma viral oncogene homolog | 20.1 |
| EGR1 | early growth response 1 | 18.1 |
| BTG2, BTG1 | BTG family, member 2; B-cell translocation gene 1, anti-proliferative | 17.6 |
| ATF3 | activating transcription factor 3 | 17.6 |
| none | GA_718609 – similar to Kruppel-like factor 2 in *Xenopus* *tropicalis* (68% identity; E-value=0.0 | 17.4 |
| JUNB | jun B proto-oncogene | 11.7 |
| NR4A1 | nuclear receptor subfamily 4, group A, member 1 | 10.1 |
| CYR61 | cysteine-rich, angiogenic inducer, 61 | 9.7 |
| DUSP1 | dual specificity phosphatase 1 | 9.5 |
| JUN | jun proto-oncogene | 7.8 |
| HES4 | hairy and enhancer of split 4 (Drosophila) | 7.2 |
| C8orf4 | chromosome 8 open reading frame 4 | 6.3 |
| DDIT4 | DNA-damage-inducible transcript 4 | 5.0 |
| ETS2 | v-ets erythroblastosis virus E26 oncogene homolog 2 (avian) | 4.3 |
| C2orf77 | chromosome 2 open reading frame 77 | 3.3 |
| C1orf51 | chromosome 1 open reading frame 51 | 2.2 |

| **Table S8.** List of transcripts which increased in Western Painted Turtle ventricle after 24 hours of anoxia at 19°C. Data are from analyses involving Log-Normal multiple comparisons model. | | |
| --- | --- | --- |
| **Human Gene**  **Ortholog** | **Gene**  **Description** | **Fold Change** |
| SLC2A14, SLC2A1,  SLC2A3 | solute carrier family 2 (facilitated glucose  transporter), members 1, 3, and 14 | 30.7 |
| FOS | FBJ murine osteosarcoma viral oncogene homolog | 20.7 |
| APOLD1 | apolipoprotein L domain containing 1 | 19.1 |
| PTGS2 | prostaglandin-endoperoxide synthase 2 (prostaglandin G/H synthase and cyclooxygenase) | 18.6 |
| JUNB | jun B proto-oncogene | 17.6 |
| BTG1,BTG2 | B-cell translocation gene 1, anti-proliferative; BTG family, member 2 | 16.6 |
| EGR1 | early growth response 1 | 12.4 |
| ATF3 | activating transcription factor 3 | 10.6 |
| CSRNP1 | cysteine-serine-rich nuclear protein 1 | 9.2 |
| DUSP1 | dual specificity phosphatase 1 | 9.1 |
| BHLHE40 | basic helix-loop-helix family, member e40 | 7.2 |
| CISH | cytokine inducible SH2-containing protein | 6.5 |
| DDIT4 | DNA-damage-inducible transcript 4 | 6.1 |
| KLF10 | Kruppel-like factor 10 | 5.9 |
| JUN | jun proto-oncogene | 5.6 |
| HES4 | hairy and enhancer of split 4 (Drosophila) | 5.6 |
| SIK1 | salt-inducible kinase 1 | 5.6 |
| CYR61 | cysteine-rich, angiogenic inducer, 61 | 5.0 |
| TIPARP | TCDD-inducible poly(ADP-ribose) polymerase | 4.9 |
| C10orf10 | chromosome 10 open reading frame 10 | 3.8 |
| NFIL3 | nuclear factor, interleukin 3 regulated | 3.3 |
| TIMM23, TIMM23B | translocase of inner mitochondrial membrane 23 homolog (yeast); translocase of inner mitochondrial membrane 23 homolog B (yeast) | 2.4 |
| RASGEF1A | RasGEF domain family, member 1A | 2.2 |

| **Table S9.** List of transcripts which decreased in Western Painted Turtle ventricle after 24 hours of anoxia at 19°C. Data are from analyses involving Log-Normal multiple comparisons model. | | |
| --- | --- | --- |
| **Human Gene**  **Ortholog** | **Gene**  **Description** | **Fold Change** |
| CDO1 | cysteine dioxygenase, type I | 0.21 |
| SRSF5 | serine/arginine-rich splicing factor 5 | 0.29 |
| LRRC53 | Leucine rich repeat containing 53 | 0.43 |
| CCNJL | cyclin J-like | 0.44 |
| MKNK1 | MAP kinase interacting serine/threonine kinase 1 | 0.50 |

**Table S10.** Percentage DNA identity between enamel and dentin pseudogenes in turtle and chicken as compared to functional crocodilian orthologs

| **AMEL** | **Turtle (***C.p. bellii***)** | **Chicken** |
| --- | --- | --- |
| *Ψ*AMELexon1 | 57.6 | 62.1 |
| *Ψ*AMELexon2 | 76.7 | 68.8 |
| *Ψ*AMELexon3 | 52.1 | 64.9 |
| *Ψ*AMELexon5 | MH | 73.3 |
| *Ψ*AMELexon6 | MH | 54.8/64.0 |
| *Ψ*AMELexon7 | 74.5 | 62.1 |
|  |  |  |
| ENAM |  |  |
| *Ψ*ENAMexon1 | 45.5 | ------ |
| *Ψ*ENAMexon4 | 54.8 | 54.7 |
| *Ψ*ENAMexon5 | 46.0 | 55.1 |
| *Ψ*ENAMexon6 | 54.8 | 51.1 |
| *Ψ*ENAMexon7 | ------ | 47.8 |
| *Ψ*ENAMexon8a | 54.2 | 67.2 |
| *Ψ*ENAMexon8b | 49.0 | 65.3 |
| *Ψ*ENAMexon9 | 50.0 | 50.0 |
| *Ψ*ENAMexon10 ^(5'-68nt)^ | 71.4 | 63.8 |
|  |  |  |
| MMP20 |  |  |
| *Ψ*MMP20exon1^†^ | 47.1 | 43.8 |
| *Ψ*MMP20exon2^†^ | 61.8 | 45.1 |
| *Ψ*MMP20exon3 | 79.9 | 45.5 |
| *Ψ*MMP20exon4 | 44.1 | 44.8 |
| *Ψ*MMP20exon5 | MH | ------ |
| *Ψ*MMP20exon6 | MH | 50 |
| *Ψ*MMP20exon7 | MH | 67.2 |
| *Ψ*MMP20exon8 | 70.7 | 44.6 |
| *Ψ*MMP20exon9 | 76.9 | 65.7 |
| *Ψ*MMP20exon10^(5'-112nt)^ | 79.5 | ------ |

† - Human sequences used

**------ =** missing exon

MH = multiple hits

**Table S11**. Percentage identity of AMBN and DSPP pseudogenes in turtle as compared to functional crocodilian orthologs.

| **AMBN** | **Turtle (***C.p. bellii***)** |
| --- | --- |
| *Ψ*AMBNexon1 | 58.9 |
| *Ψ*AMBNexon2 | 82.6 |
| *Ψ*AMBNexon3 | 70.6 |
| *Ψ*AMBNexon4 | 50.0 |
| *Ψ*AMBNexon5 | 47.5 |
| *Ψ*AMBNexon6 | 56.5 |
| *Ψ*AMBNexon7 | 64.3 |
| *Ψ*AMBNexon10 | 67.2 |
| *Ψ*AMBNexon11 | 66.7 |
| *Ψ*AMBNexon12 | 64.6 |
| *Ψ*AMBNexon13 | 39.5 |
|  |  |
| **DSPP** |  |
| *Ψ*DSPPexon1 | 48.0 |
| *Ψ*DSPPexon2 | 59.3 |
| *Ψ*DSPPexon3 | **------** |
| *Ψ*DSPPexon4 | **------** |
| *Ψ*DSPPexon5 | **------** |

**------ =** missing exons

**Table S12.** Results of exon-wise BLAST search and percentage identity between selected aging associated genes against genomes of three turtles, using sequences from the anole lizard (A) or chicken (C) as query.

|  | *Chrysemys p. bellii* | *Trachemys scripta* | *Apalone spinifera* |
| --- | --- | --- | --- |
| TEP1 (A) | No hits | No hits | No hits |
| POT1 (C) | No hits | No hits | 86% (exons 10 & 22), 85% (exons 15 & 27) |
| TERF2IP (C) | No hits | No hits | No hits |
| ATP5O (C) | No hits | No hits | No hits |
| PLCG2 (A) | 91% (exons 10 & 30) 88% (exon 12), 85% (exon 16) | 90% (exon 10), 85% (exon 16), 91% (exon 30) | 86% (exon 1), 89% (exon 10), 88% (exon 12), 87% (exon 16), 90% (exon 30) |

**Table S13.** GC content of the coding and upstream regulatory region of Western Painted Turtle genes known to underlie gonadal development in vertebrates or linked to sex chromosomes in chicken.

|  | **CDS** | | | **20kb Upstream** | | |  |
| --- | --- | --- | --- | --- | --- | --- | --- |
| **Gene** | **Complete CDS?** | **CDS Length (bp)** | **CDS GC Content (%GC)** | **Gap Length (bp)** | **Upstream Length (bp)** | **Upstream GC Content (%GC)** | **CDS/**  **Upstream** |
| 5a-Reductase | Y | 768 | 52.6 | 1310 | 18690 | 47.6 | 1.11 |
| Aco1 | Y | 2670 | 48.7 | 9665 | 10335 | 42.8 | 1.14 |
| AMH | Y | 2037 | 61.4 | 480 | 19520 | 47.7 | 1.29 |
| AR | Y | 2373 | 60.3 | 4942 | 15058 | 44.6 | 1.35 |
| Aromatase | Y | 1512 | 42.1 | 0 | 20000 | 43.5 | 0.97 |
| ATP5A1 | Y | 1662 | 48.9 | 9227 | 10773 | 45.0 | 1.09 |
| Chd1 | Y | 5169 | 41.7 | 907 | 19093 | 44.0 | 0.95 |
| Ctnnb1 | Y | 2346 | 48.3 | 3130 | 16870 | 41.7 | 1.16 |
| Dax1 | N | 797 | 63.0 | 670 | 19330 | 43.7 | 1.44 |
| Dmrt1 | Y | 1107 | 53.6 | 0 | 20000 | 41.8 | 1.28 |
| Dmrt2 | Y | 1563 | 51.7 | 0 | 20000 | 44.1 | 1.17 |
| Dmrt3 | Y | 1365 | 54.0 | 0 | 9838 | 40.7 | 1.33 |
| Emx2 | Y | 744 | 55.5 | 94 | 19906 | 41.8 | 1.33 |
| ER alpha | Y | 1767 | 48.6 | 0 | 20000 | 41.4 | 1.17 |
| ER beta | Y | 1671 | 41.7 | 4573 | 15427 | 39.3 | 1.06 |
| Fgf9 | Y | 627 | 45.3 | 291 | 19709 | 43.4 | 1.04 |
| Fhl2 | Y | 840 | 46.9 | 0 | 20000 | 42.8 | 1.10 |
| Fog2 | Y | 3456 | 43.9 | 1462 | 18538 | 39.8 | 1.10 |
| Foxl2 | Y | 906 | 66.0 | 6000 | 14000 | 41.0 | 1.61 |
| Gata2 | Y | 1404 | 58.8 | 1472 | 18528 | 48.8 | 1.20 |
| Gata4 | Y | 1197 | 63.7 | 10000 | 10000 | 44.8 | 1.42 |
| GHR | Y | 1848 | 44.3 | 20 | 19980 | 41.3 | 1.07 |
| Gpn3 | Y | 855 | 40.7 | 2455 | 17545 | 42.5 | 0.96 |
| Lhx9 | Y | 1203 | 52.0 | 0 | 20000 | 39.0 | 1.33 |
| Lim1 | Y | 2118 | 46.8 | 6000 | 14000 | 33.0 | 1.42 |
| Myst2 | Y | 1860 | 48.9 | 3752 | 16248 | 43.7 | 1.12 |
| Ptch1 | Y | 4341 | 46.9 | 550 | 19450 | 44.7 | 1.05 |
| Rps6 | Y | 750 | 48.8 | 95 | 19905 | 44.3 | 1.10 |
| Rspo1 | Y | 783 | 52.1 | 0 | 20000 | 51.0 | 1.02 |
| Sf1 | Y | 1401 | 55.3 | 20 | 19980 | 55.9 | 0.99 |
| Sox9 | Y | 1479 | 59.3 | 9055 | 10945 | 39.0 | 1.52 |
| Tubg1 | Y | 1356 | 58.4 | 739 | 19261 | 46.7 | 1.25 |
| Wnt4 | Y | 1056 | 55.6 | 786 | 19214 | 48.3 | 1.15 |
| Wt1 | Y | 1254 | 54.7 | 1433 | 18567 | 41.6 | 1.31 |
| **Average** |  |  | 51.8 |  |  | 43.6 | 1.19 |
| **STDEV** |  |  | 6.9 |  |  | 4.0 | 0.17 |

**Table S14.** Presence and putative location of immune-related genes in the Western Painted Turtle.

| Gene | Pre-Site Scaffold | cDNA | Predicted Gene |
| --- | --- | --- | --- |
| **B cells and APCs** | | | |
| BLNK | JH584723.1 | Cp.145650.1 | 1438436 |
| CD226 | JH584442.1 |  | 605915 |
| CD40 | JH584774.1 |  | 541981 |
| CD40L | JH584720.1 |  | 1277473 |
| CD79 | JH585853.1 |  |  |
| IgJ | JH584761.1 |  | 565875 |
| IgM_constant | JH584564.1 |  | 3883 |
| PIgR | JH584805.1 |  | 495325 |
| PTPRC | JH584765.1 |  | 1265501 |
| **Chemokines and receptors** | | | |
| CCR5 | JH584392.1 | Cp.55826.1 |  |
| CCR6 | JH584758.1 |  | 607329 |
| CCR7 | JH584629.1 |  | 525789 |
| CCR9 | JH584392.1 |  | 566240 |
| CXCR1 | JH584422.1 |  |  |
| CXCR2 | JH584422.1 |  |  |
| CXCR3 | JH584551.1 |  |  |
| CXCR4 | JH584514.1 | Cp.8949.1 |  |
| **Complement cascade** | | | |
| C1 | JH585044.1 |  | 52873 |
| C3 | JH585097.1 |  |  |
| C4 | JH584789.1 |  | 593426 |
| C5 | JH584698.1 | Cp.115157.2 | 496952 |
| C6 | JH584451.1 |  | 588353 |
| C7 | JH584451.1 |  | 588351 |
| C8 | JH584487.1 | Cp.79161.1 | 563251 |
| C9 | JH584451.1 |  | 67313 |
| **Interleukins and interferons** | | | |
| FOXP3 | JH584856.1 |  | 9148 |
| IFN-γ | Contig298521.1 |  |  |
| IKBKG | JH584428.1 |  |  |
| IL-1B | Contig200152.1 |  |  |
| IL-2RB | JH584735.1 |  |  |
| IL-2RG | JH584503.1 |  | 1835889 |
| IL-6 | JH584577.1 |  |  |
| IL-6ST | JH584810.1 | Cp.111479.2 | 588097 |
| IL-8 | JH584761.1 |  | 566057 |
| IL-17D | JH584766.1 | Cp.94956.1 |  |
| IL-17RA | JH584531.1 |  | 638066 |
| IL-17RD | JH584925.1 |  | 32865 |
| IL-22 | JH584425.1 |  | 561521 |
| IL-27 | JH584766.1 | Cp.94956.2 |  |
| MAVS | Contig261453.1 |  |  |
| MYD88 | JH584683.1 |  |  |
| TGFβ | JH584944.1 | Cp.142591.1 |  |
| TNFα | JH584606.1 | Cp.38020.1 | 588418 |
| **MHCI pathway** | | | |
| ERAP1 | JH584476.1 |  | 103786 |
| ERAP2 | JH584476.1 |  |  |
| FAS (CD178) | Contig361712.1 |  |  |
| FASL (CD95) | JH584764.1 |  | 1266007 |
| GranzymeB | JH585438.1 |  | 660924 |
| Perforin | JH584629.1 |  |  |
| PSMB1 | JH584581.1 | Cp.30760.1 | 564975 |
| PSMB2 | JH584691.1 | Cp.98576.1 | 1295977 |
| PSMB3 | JH584731.1 | Cp.151225.1 | 496982 |
| PSMB4 | JH584619.1 | Cp.42396.1 | 598525 |
| PSMB5 | JH584564.1 | Cp.56439.1 | 5513 |
| PSMB6 | AHGY01427850.1 | Cp.130941.1 |  |
| PSMB7 | JH584413.1 | Cp.44009.2 | 495792 |
| PSMB8 | JH584419.1 | Cp.56439.1 | 5513 |
| PSME1 | JH584564.1 | Cp.25323.1 |  |
| PSME2 | JH584564.1 | Cp.25261.1 | 661151 |
| PSME3 | JH584926.1 | Cp.138127.1 | 641856 |
| RFXANK | JH584533.1 | Cp.16841.2 | 621075 |
| TAP1 | JH584419.1 |  | 26369 |
| TAPBP | JH585206.1 |  |  |
| UNC-93B | JH584650.1 |  | 61960 |
| **MHCII pathway** | | | |
| CIITA | JH584814.1 |  | 914717 |
| Invariant chain | JH584667.1 |  | 645737 |
| MHC II α | JH586679.1 |  | 1916502 |
| MHC II β | AHGY01282570.1 |  | 1272057 |
| RFX5 | JH584619.1 |  | 61608 |
| RFX7 | JH584631.1 | Cp.44936.2 | 640772 |
| RFXAP | JH584512.1 |  | 603129 |
| **T-cell receptors (TcR)** | | | |
| AICDA (AID) | JH584665 |  | 136690 |
| AIRE | JH584990.1 |  | 758647 |
| CD247 | JH584471.1 |  | 589327 |
| CD3γ | JH584702.1 |  |  |
| CD3δ | JH584702.1 |  |  |
| CD3ε | JH584702.1 |  |  |
| CD4 | JH584582.1 |  |  |
| CD8α | AHGY01414786.1 |  | 95811 |
| RAG1 | JH584770.1 |  | 781599 |
| RAG2 | JH584770.1 |  | 552046 |
| TCRα | Contig116991.1 |  |  |
| **TLR-pathway** | | | |
| TLR1a | JH584508.1 |  | 1821888 |
| TLR1b | JH584508.1 | Cp.6521.1 | 1819961 |
| TLR2 | JH584697.1 | Cp.113618.1 |  |
| TLR3 | JH584449.1 | Cp.66554.1 |  |
| TLR4 | JH584724.1 | Cp.45031.1 | 541962 |
| TLR5 | JH584552.1 |  | 559658 |
| TLR6 | Contig99868.1 |  |  |
| TLR7 | JH584528.1 |  | 592299 |
| TLR8 | Contig159833.1 |  |  |
| TLR9 | JH585269.1 |  |  |
| TLR14 | JH584619.1 |  |  |
| TLR15 | JH584831.1 |  |  |
| TLR21 | JH585853.1 |  |  |

**Table S15.** Toll-like Receptor (TLR) representation in vertebrates.

| **TLR** | **Mammal**  *Homo sapiens*^1^ | **Mammal**  *Mus musculus*^1^ | **Bird**  *Gallus gallus*^1^ | **Amphibian**  *Xenopus tropicalis*^1^ | **Fish**  *Danio rerio*^1^ | **Reptile**  *Anolis carolinensis*^2^ | **Reptile**  *Chrysemys picta* |
| --- | --- | --- | --- | --- | --- | --- | --- |
| 1 | + | + | +^3^ | +^3^ | + | - | +^3^ |
| 2 | + | + | + ^3^ | + | + | + | + |
| 3 | + | + | + | + | + | + | + |
| 4 | + | + | + | + | +^3^ | + | + |
| 5 | + | + | - | + | +^3^ | + | + |
| 6 | + | + | - | - | - | + | + |
| 7 | + | + | + ^3^ | + | + | + | + |
| 8 | + | - | + | + | +^3^ | - | + |
| 9 | + | + | - | + | + | - | + |
| 10 | + | - | - | - | - | - | - |
| 11 | - | + | - | + | - | - | - |
| 12 | - | + | - | - | - | - | - |
| 13 | - | + | - | + | - | + | - |
| 14 | - | - | - | +^3^ | + | - | + |
| 15 | - | - | + | - | - | - | + |
| 16 | - | - | - | + | - | - | - |
| 18 | - | - | - | - | + | - | - |
| 19 | - | - | - | - | - | - | - |
| 20 | - | - | - | - | +^3^ | - | - |
| 21 | - | - | + | + | - | - | + |
| 22 | - | - | - | + | + | - | - |

^1^ Leulier and Lemaitre et al 2008

^2^ Ensembl

^3^ Gene has been expanded.

**Table S16.** Number of predicted gene models in *Chrysemys p. bellii.* Gene quality indices (gene, pseudogene, fragment) are based on (Heger et al. 2007), and are defined as follows: **Gene**- alignment between template and prediction cover more than 80% of the template, no in-frame stop codons and frame-shifts ; **Pseudogene**- prediction contains frame-shifts and disruptions. Frame-shifts and disruptions are allowed in dubious exons, with exceptionally low sequence similarity to the template when compared to other exons in the transcript; **Fragment**- alignment between template and prediction covers less than 80% of the template.

| Type | number |
| --- | --- |
| **Genes** |  |
| CG (conserved genes) | 8583 |
| PG (partially conserved genes) | 9735 |
| SG (single exon genes) | 3478 |
| RG (retrotransposed genes) | 263 |
| UG (not conserved genes) | 1096 |
|  |  |
| **Pseudogenes** |  |
| CP (duplicated pseudogene) | 442 |
| SP (single exon pseudogene) | 2303 |
| PP (partially conserved pseudogene) | 5263 |
| RP (processed pseudogene) | 277 |
| UP (not conserved pseudogene) | 1795 |
|  |  |
| **Fragments** |  |
| SF (single exon fragment) | 937 |
| CF (conserved fragment) | 28 |
| PF (partially conserved fragment) | 4679 |
| UF (not conserved fragment) | 614 |
| BF (pseudogenic fragment) | 2026 |

**Table S17.** Number of genes in each species belonging to a protein coding family that expanded in a lineage specific manner in *C. picta*. Lineage specific gene expansions are identified relative to the last common ancestor; some families such as the immunoglobulin like receptors or the beta-keratins show independent expansions in different lineages. These gene families are listed in the same order as Figure 6.

| family | human | mouse | platypus | chicken | zebrafinch | Painted Turtle | Green anole | tetraodon | Zebrafish |
| --- | --- | --- | --- | --- | --- | --- | --- | --- | --- |
| SANT | 0 | 0 | 0 | 0 | 0 | 355 | 0 | 0 | 0 |
| Immunoglobulin heavy chain | 8 | 2 | 49 | 33 | 3 | 325 | 25 | 18 | 0 |
| Pre-B lynphocyte | 2 | 7 | 42 | 10 | 6 | 131 | 8 | 2 | 5 |
| beta-keratin | 85 | 102 | 6 | 124 | 133 | 106 | 6 | 2 | 2 |
| Immunoglobulin kappa chain | 9 | 5 | 8 | 0 | 0 | 94 | 3 | 0 | 4 |
| C-type lectin domain family | 18 | 24 | 66 | 15 | 6 | 73 | 30 | 5 | 2 |
| SCAN | 0 | 0 | 0 | 0 | 0 | 43 | 1 | 0 | 0 |
| Scavenger receptor cys-rich | 10 | 7 | 22 | 18 | 5 | 42 | 11 | 2 | 1 |
| Zinc finger protein 451 | 2 | 1 | 2 | 1 | 1 | 41 | 17 | 1 | 1 |
| Peptidase / granzyme | 12 | 23 | 6 | 5 | 5 | 38 | 5 | 3 | 14 |
| Killer cell immunoglobulin-like receptor | 21 | 16 | 4 | 109 | 1 | 35 | 1 | 1 | 12 |
| Killer cell lectin-like receptor subfamily | 3 | 6 | 10 | 3 | 1 | 27 | 4 | 0 | 1 |
| EMR | 3 | 2 | 3 | 0 | 0 | 26 | 1 | 1 | 10 |
| Cytochrome P450 | 17 | 42 | 10 | 9 | 8 | 19 | 43 | 3 | 3 |
| Solute carrier family 22 | 3 | 4 | 6 | 1 | 1 | 18 | 7 | 1 | 1 |
| ribonuclease | 9 | 16 | 3 | 2 | 1 | 18 | 8 | 0 | 1 |
| Alpha / beta hydrolase | 7 | 22 | 7 | 4 | 5 | 12 | 11 | 6 | 1 |
| butyrophilin | 0 | 0 | 0 | 0 | 0 | 12 | 0 | 0 | 0 |
| NLR family, pyrin domain containing | 5 | 4 | 4 | 1 | 1 | 10 | 2 | 0 | 3 |
| ficolin | 3 | 2 | 1 | 1 | 1 | 9 | 3 | 1 | 1 |
| Fatty acid-binding protein | 3 | 3 | 1 | 3 | 3 | 8 | 3 | 1 | 1 |
| Upregulator of cell proliferation | 1 | 1 | 0 | 0 | 1 | 7 | 0 | 0 | 3 |
| HEPACAM | 0 | 0 | 0 | 2 | 0 | 7 | 0 | 0 | 0 |
| Zinc finger, BED-type containing | 1 | 0 | 0 | 0 | 0 | 7 | 1 | 0 | 0 |
| interleukin | 1 | 2 | 1 | 1 | 1 | 5 | 2 | 2 | 1 |
| Cathepsin S | 1 | 1 | 1 | 1 | 1 | 4 | 1 | 1 | 2 |
| gallinacin | 0 | 0 | 0 | 2 | 2 | 4 | 0 | 0 | 0 |
| Ceroid-lipofuscnosis, neuronal | 1 | 1 | 0 | 1 | 1 | 4 | 0 | 0 | 1 |
