# Supplementary material for: The western painted turtle genome, a model for the evolution of extreme physiological adaptations in a slowly evolving lineage
Source: Genome Biol. 2013 Mar 28;14(3):R28. doi: 10.1186/gb-2013-14-3-r28 (PMC4054807; doi:10.1186/gb-2013-14-3-r28)
Supplement: Additional file 2 — Supplementary figures. Figures S1-S12 contain additional information in support of the painted turtle repeat analyses (Figures S1-S6), isochores (Figures S7-S9), and anoxia tolerance (Figures S10-12). [file gb-2013-14-3-r28-S2.DOCX]

**Supplementary Figures**

**
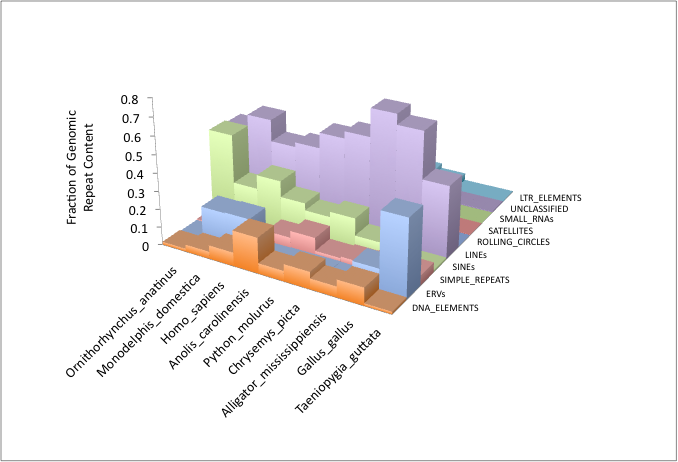
Figure S1.** The fraction of global genomic repeat content plotted for high abundance repeat classes among exemplar genome assemblies representing all major amniote clades.

**
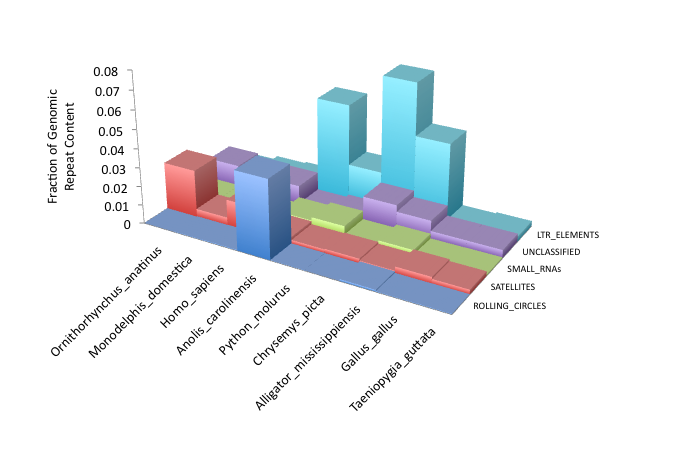
Figure S2.** The fraction of global genomic repeat content plotted for low abundance repeat classes among exemplar genome assemblies representing all major amniote clades.

**
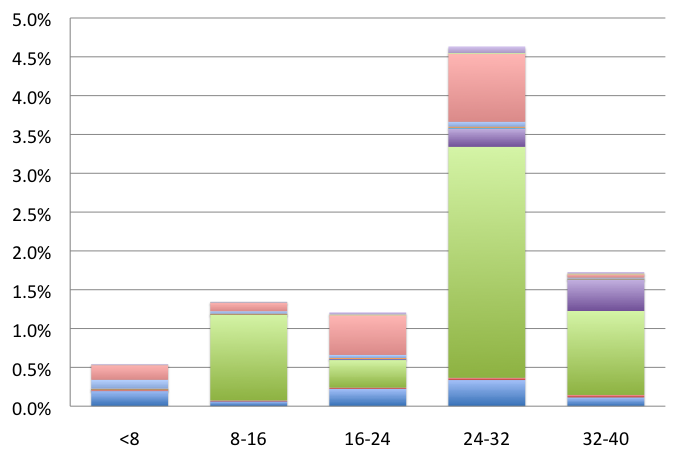

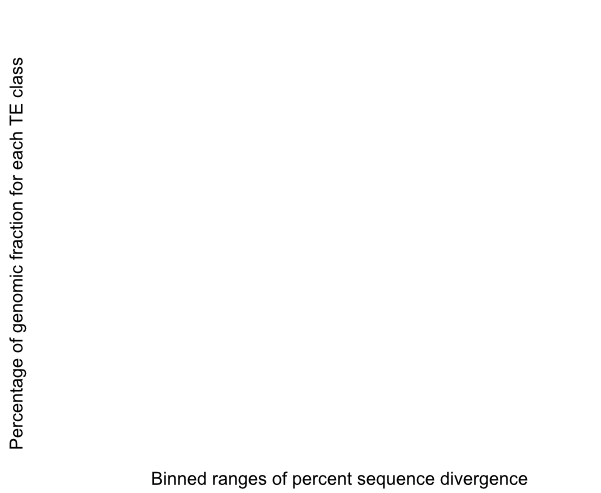
**

**Figure S3.** Stacked histogram of the distribution of sequence divergence values for major classes of genomic repeats in the Western Painted Turtle genome assembly. Y axis values are percentages of the genomic fractions for each class of repeat. X axis values are the ranges of percent sequence divergence based on RepeatMasker annotation.
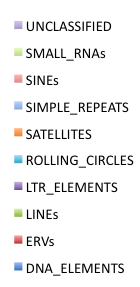


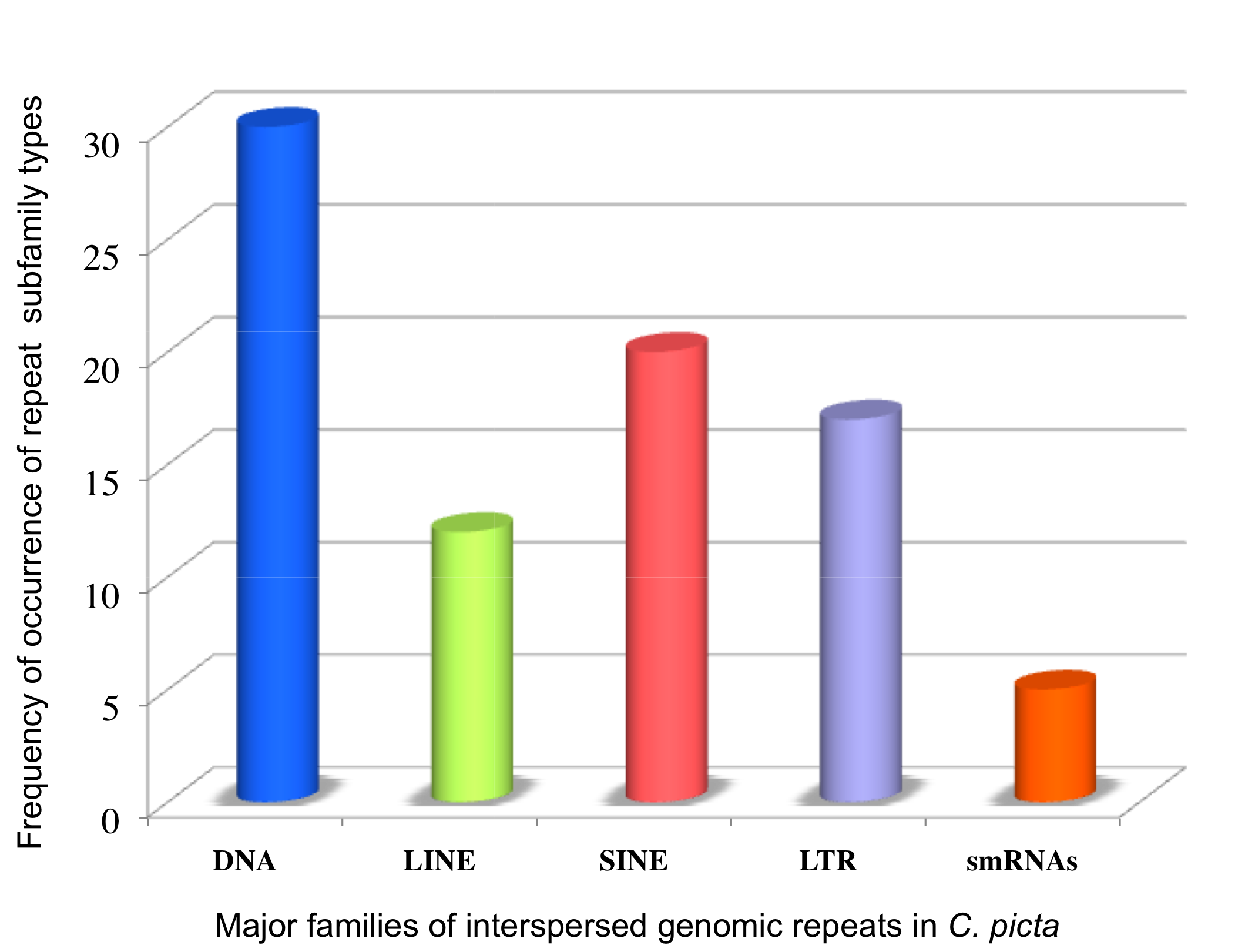


**Figure S4.** Frequency of occurrence of different *C. picta* subfamily types within major TE classes, based on RepeatMasker annotation output.

**Figure S5.** Divergence distributions of TEs in four exemplar amniote genome assemblies: Alligator: *Alligator mississipiensis*, Anolis: *Anolis carolinensis,* Zebra Finch: *Taeniopygia guttata,* Human: *Homo sapiens.* Y axis values are percentages of the genomic fractions for each class of repeat. X axis values are the ranges of percent sequence divergence based on RepeatMasker annotation.

**
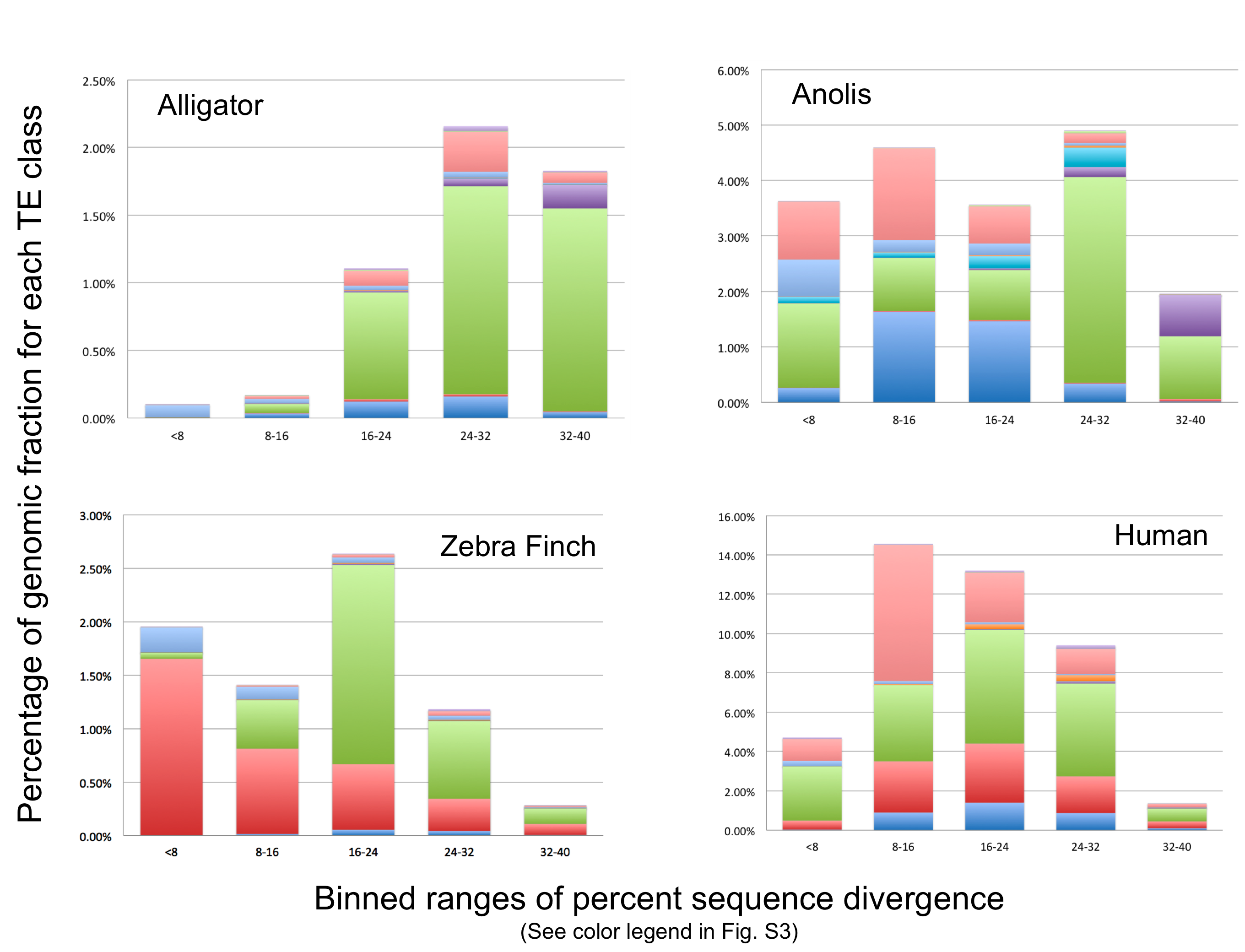
**

**
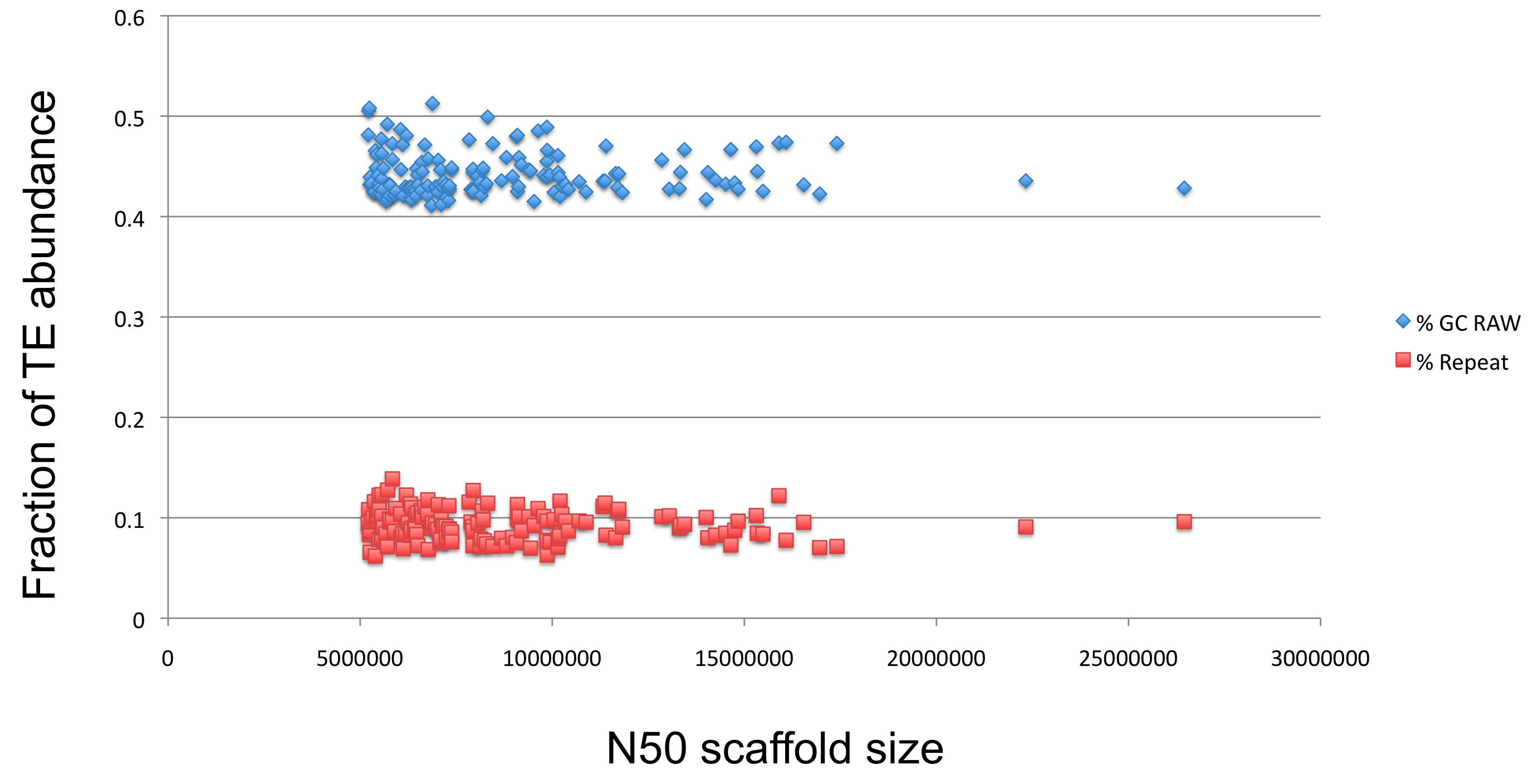
**

**Figure S6.** Percentages of TE abundance vs. G+C plotted by N50 scaffold size for the *C. picta* assembly.**Figure S7.** Distributions of GC content in amniote genomes based on 3-kb nonoverlapping windows.

**
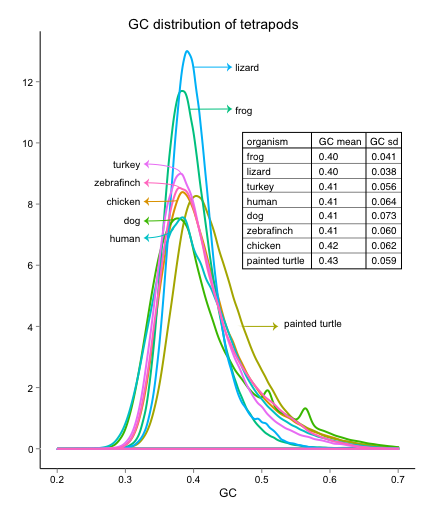
**

**Figure S8.** Genic GC (as measured by GC3) exhibits a weak but significant correlation with its flanking GC content (10-kb on each side), a pattern more in line with the lizard genome than with human or chicken. N=11,280 genes.


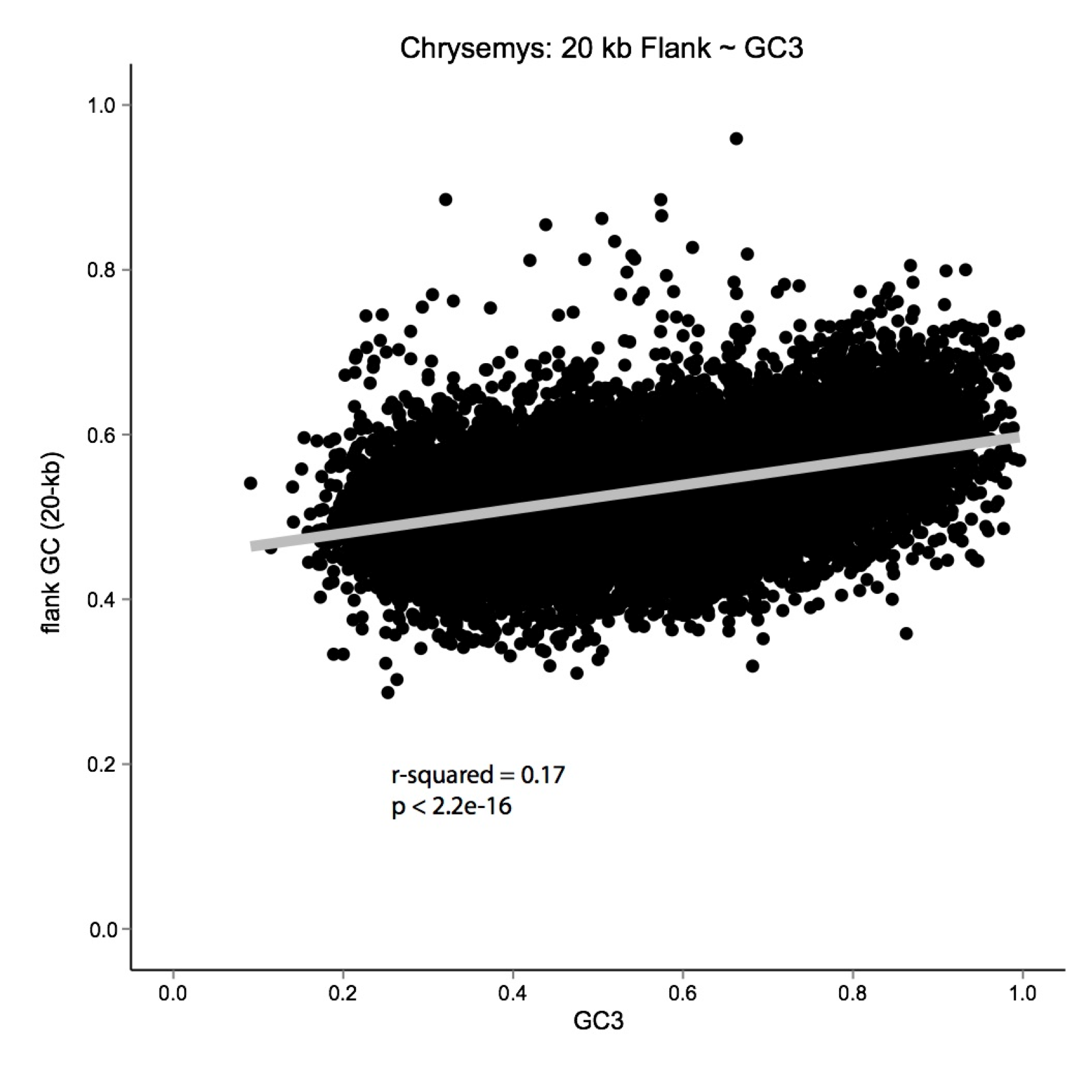


**Figure S9.** Amniote phylogeny with branch lengths proportional to Di,j (the divergence of GC3 between two nodes of a branch).  The current GC3 and the equilibrium GC3* are also shown and demonstrate the expected continued trajectory of GC content evolution.**Figure S10.** RNA-Seq read depths across three statistically upregulated genes in eight telencephalon samples. The x-axis shows the loci of the reads aligned using TopHat.


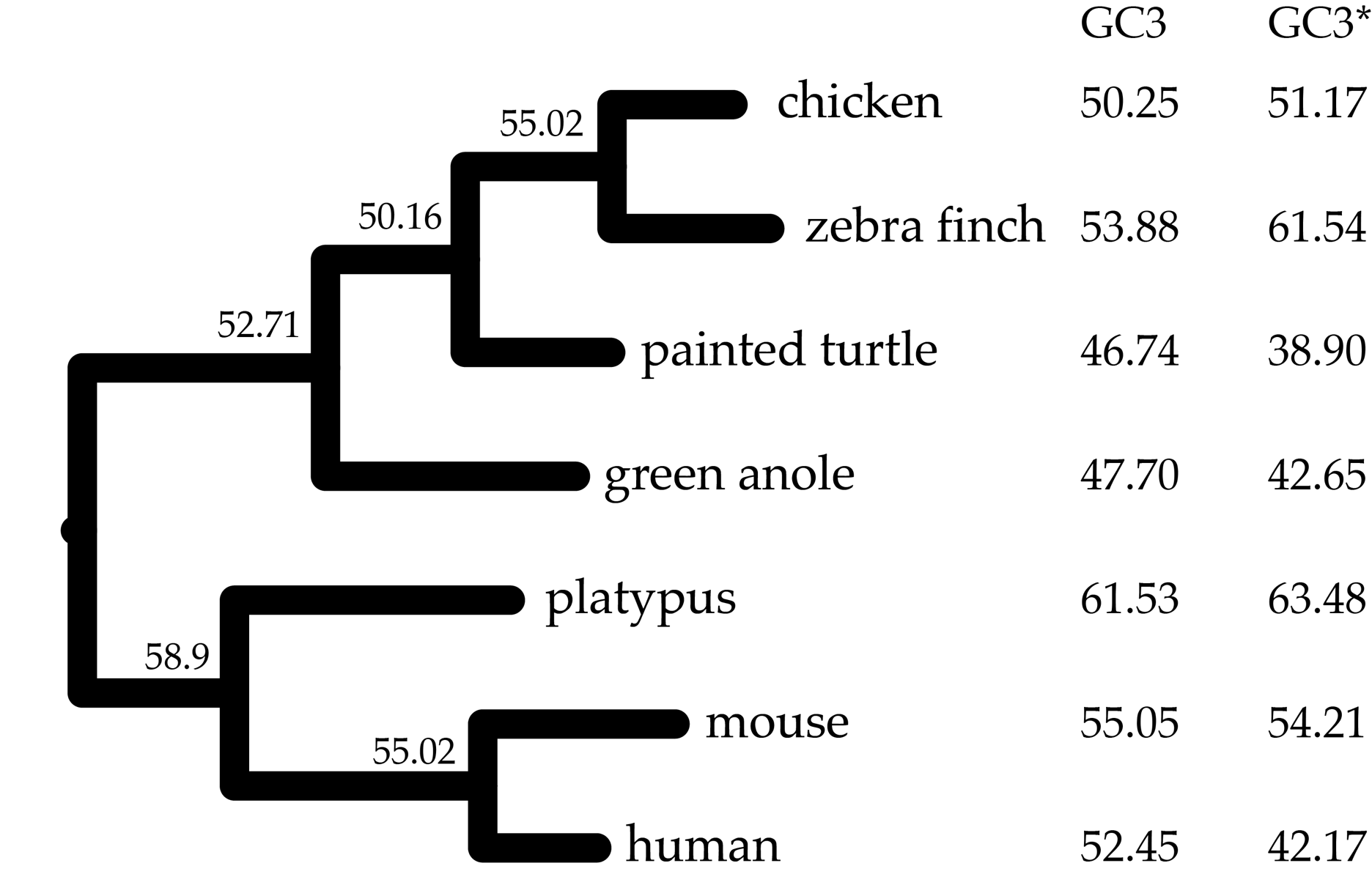

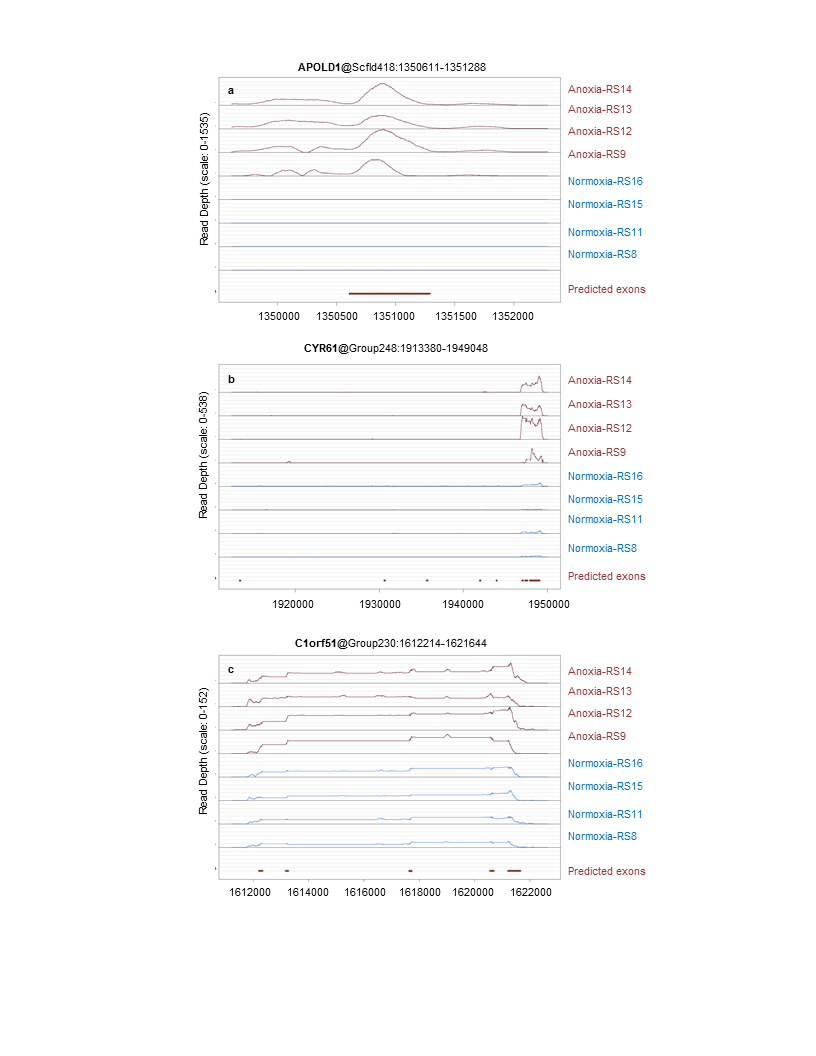


**Figure S11.** RNA-Seq read depths across three statistically upregulated genes in 8 ventricular samples. The x-axis shows the loci of the reads aligned using TopHat.

**Figure S12.** RNA-Seq read depths across three statistically downregulated genes in 8 ventricular samples. The x-axis shows the loci of the reads aligned using TopHat.


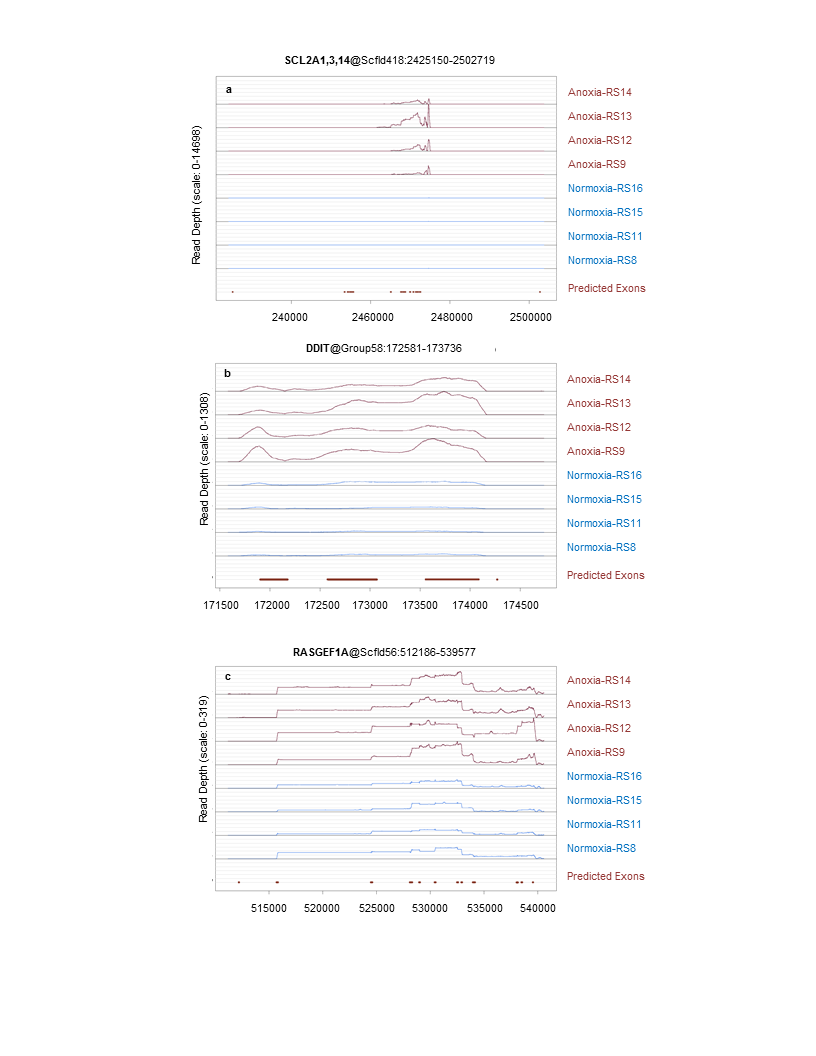


**
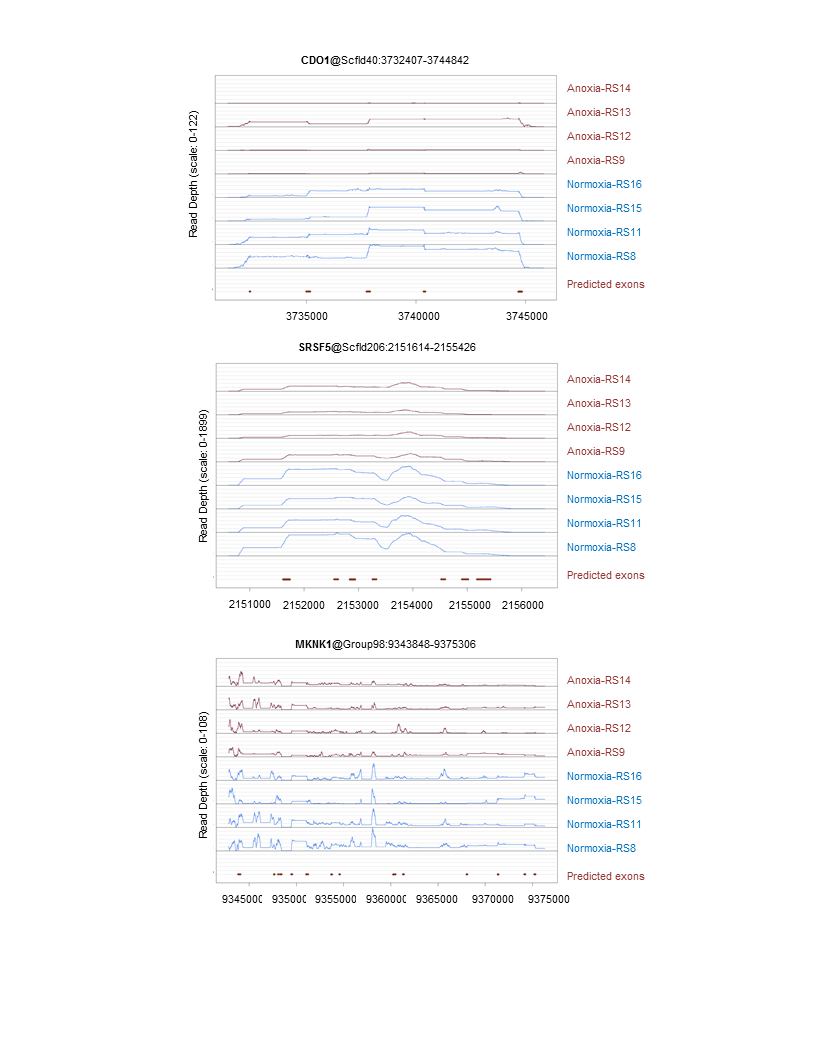
**
